# A shift from motorised travel to active transport: What are the potential health gains for an Australian city?

**DOI:** 10.1371/journal.pone.0184799

**Published:** 2017-10-11

**Authors:** Belen Zapata-Diomedi, Luke D. Knibbs, Robert S. Ware, Kristiann C. Heesch, Marko Tainio, James Woodcock, J. Lennert Veerman

**Affiliations:** 1 The University of Queensland, School of Public Health, Brisbane, Queensland, Australia; 2 Griffith University, Menzies Health Institute Queensland, Brisbane, Queensland, Australia; 3 Queensland University of Technology, Institute of Health & Biomedical Innovation and the School of Public Health and Social Work, Brisbane, Queensland, Australia; 4 MRC Epidemiology Unit & UKCRC Centre for Diet and Activity Research (CEDAR), University of Cambridge School of Clinical Medicine, Cambridgeshire, Cambridge, United Kingdom; 5 Systems Research Institute, Polish Academy of Sciences, Mazovia, Warsaw, Poland; 6 Cancer Council NSW, Sydney, New South Wales, Australia; University of Washington, UNITED STATES

## Abstract

**Introduction:**

An alarmingly high proportion of the Australian adult population does not meet national physical activity guidelines (57%). This is concerning because physical inactivity is a risk factor for several chronic diseases. In recent years, an increasing emphasis has been placed on the potential for transport and urban planning to contribute to increased physical activity via greater uptake of active transport (walking, cycling and public transport). In this study, we aimed to estimate the potential health gains and savings in health care costs of an Australian city achieving its stated travel targets for the use of active transport.

**Methods:**

Additional active transport time was estimated for the hypothetical scenario of Brisbane (1.1 million population 2013) in Australia achieving specified travel targets. A multi-state life table model was used to estimate the number of health-adjusted life years, life-years, changes in the burden of diseases and injuries, and the health care costs associated with changes in physical activity, fine particle (<2.5 μm; PM_2.5_) exposure, and road trauma attributable to a shift from motorised travel to active transport. Sensitivity analyses were conducted to test alternative modelling assumptions.

**Results:**

Over the life course of the Brisbane adult population in 2013 (860,000 persons), 33,000 health-adjusted life years could be gained if the travel targets were achieved by 2026. This was mainly due to lower risks of physical inactivity-related diseases, with life course reductions in prevalence and mortality risk in the range of 1.5%-6.0%. Prevalence and mortality of respiratory diseases increased slightly (≥0.27%) due to increased exposure of larger numbers of cyclists and pedestrians to fine particles. The burden of road trauma increased by 30% for mortality and 7% for years lived with disability. We calculated substantial net savings ($AU183 million, 2013 values) in health care costs.

**Conclusion:**

In cities, such as Brisbane, where over 80% of trips are made by private cars, shifts towards walking, cycling and public transport would cause substantial net health benefits and savings in health care costs. However, for such shifts to occur, investments are needed to ensure safe and convenient travel.

## Introduction

The built environment, largely determined by policies in the planning and transport sectors, contributes greatly to health risk factors including physical inactivity, traffic-related air pollution and road trauma globally [1, 2].

An emerging body of literature is examining the impacts of built environment initiatives on health and economic outcomes. Two recent reviews of economic evaluation and health impact assessments of active transport interventions found that the greatest individual health gains of active travel policies are achieved by increasing physical activity (PA) levels [3, 4]. For those who undertake active travel, trade-offs can arise in terms of higher exposure to environmental hazards, including pollution (air and noise), heat and road injuries [4, 5], but the evidence suggests that PA benefits outweigh these other risks [4, 6, 7].

In Australia, 57% of adults do not meet national PA guidelines [8], and inactivity has been estimated to result in the annual loss of nearly 124,000 disability-adjusted life years in 2015 (DALYs) [9]. With nearly 80% of adults’ travel for work or education by private cars [10], encouraging active travel is a feasible strategy for improving population health. Government and non-government agencies are working towards a shift from private cars towards active transport [11–15]. For Brisbane, the capital of the State of Queensland and Australia’s third most populous city, strategic planning at the city and state level aims to achieve a mode share of 15% for walking, 5% for cycling and 14% for public transport [11, 13]. Quantifying health outcomes of Brisbane’s transport strategy could support the case for the required investment.

In this study, we quantified health outcomes and health care costs of replacing private car trips with active transport in Brisbane. A shift from car travel to active transport was based on the transport targets for South East Queensland [11] and Brisbane [13] We projected the potential health benefits and health care cost savings of a linear annual increase in active travel at the expense of private car travel from 2013 to fully achieving the targets in 2026.

## Methods

### Study area

The Brisbane Local Government Area is located in South East Queensland, Australia. Brisbane consists of 188 mainland suburbs as well as additional islands and localities in Moreton Bay [16]. A total of 1,131,191 people lived in Brisbane in 2013, with over 80% of the population aged over 15 years (median 34.5 years) [17, 18].

### Survey data

We used daily travel information collected from the South East Queensland Household Travel survey [19]. Data were collected from April to September 2009 [20]. Data from the 2012 version of the survey were available, but were not used because the survey was incomplete [21].

For the survey, the sampling unit was at the household level, and data were collected on all individuals (≥5 years of age) living in selected households. Information about all trips taken in a one week period was self-reported via paper-based questionnaire. Households were selected using a three-stage, variable proportion, clustered sampling of household addresses within Census Collection Districts (CCDs). CCDs are the second smallest geographical collection unit for census and population data collection and processing [22]. Sampling regions were divided into those participating and not participating in the Travel Smart program, a behaviour change program that aimed to reduce private car travel by increasing active transport and share rides and that was being implemented at the time of data collection [11]. The sampling process consisted of randomly selecting CCDs in each sampling region, followed by a random selection of 56 dwellings within each CCD, with 42 of them kept in the primary sample and 14 as potential replacements [20]. Field checks took place for the identification of sample loss and to ensure that at least 42 dwellings per CCD would be included.

In total 4,240 households in Brisbane responded to the survey, representing a response rate of 52%, 11,191 persons and 32,536 trips [20]. As the sample of selected households may not have been representative of Brisbane households, population weights were applied to the trip and person level [20]. Trips that were made by persons aged <17 years; were taken on weekends; or did not include walking, cycling, public transport or private car were excluded, leaving 19,385 trips available for analysis. Analyses were conducted using Stata v.13 (*StataCorp*, *College Station*, *TX)*. A trip was defined as a one-way movement from one place to another with a single purpose (transport or recreational). One trip could include multiple modes [20]. Two variables were provided in the data set to represent the main mode for a multi-modal trip: (1) longest-time mode; and (2) priority mode based on hierarchical order for public transport [20]. The longest-time mode assigned the main mode for a multi-modes trip to the mode used with the longest time. These variables were highly correlated (r = 0.99). We used the longest-time mode variable given that no justification was given in the source document for the chosen hierarchy.

### Travel patterns

Of all weekday trips made by Brisbane adults in 2009, 24% were <2km, 24% 2-5km, 33% 6–16 km and 19% >17 km. Our estimates for trips <5 km are higher than those reported for Australia as a whole (25% [10]). However, national census data refer to trips for work or full-time study only, whereas we included all transport trips. Census data stated that for Brisbane, 24% of trips for work commutes were < 5 km and 22% were 5–10 km [23]. Similarly, in our dataset 21% of work commute trips were <5 km and 23% were 5–10 km. Table 1 depicts the 2009 average number of weekday trips in total and by mode, by age and sex.

**Table 1:** Mode-specific mean (95% uncertainty interval (UI)) trips per weekday in 2009, by age and sex.

Age (years) and sex	Car occupant	Walk	Bicycle	Public Transport	Total
17–49, male	2.07 (1.94 to 2.19)	0.22 (0.18 to 0.27)	0.063 (0.038 to 0.087)	0.28 (0.23 to 0.33)	2.71 (2.59 to 2.83)
17–49, female	2.80 (2.66 to 2.95)	0.33 (0.28 to 0.38)	0.018 (0.009 to 0.026)	0.29 (0.25 to 0.33)	3.46 (3.32 to 3.60)
50–74, male	2.51 (2.37 to 2.68)	0.25 (0.19 to 0.31)	0.031 (0.012 to 0.05)	0.13 (0.09 to 0.16)	2.96 (2.79 to 3.13)
50–74, female	2.38 (2.22 to 2.54)	0.27 (0.31 to 0.33)	0.004 (-0.002 to 0.010)	0.18 (0.14 to 0.23)	2.85 (2.70 to 3.02)
75 plus, male	1.81 (1.45 to 2.18)	0.14 (0.06 to 0.22)	-	0.09 (0.015 to 0.16)	2.07 (1.72 to 2.42)
75 plus, female	1.17 (0.87–1.45)	0.13 (0.05–0.20)	-	0.15 (0.06–0.24)	1.48 (1.18–1.77)

### Travel targets

Brisbane aims for a travel mode share of 15% for walking and 5% for cycling [13]. Because no targets were proposed for public transport, we applied the regional (South East Queensland) target of a 14% share [11]. At the regional level, similar targets were proposed for walking and cycling, with an overall share of 20% for active transport. We interpreted the targets to apply only to adults, given that separate targets were proposed at the regional level for trips to school (made by children) [11]. We assumed that the targets apply to weekday trips, given that this was specified at the regional level [11]. We followed the South East Queensland report that outlined the travel targets [11], and which recommended that trips <5 km can be made by bicycle. The Queensland Government Department of Transport and Main Roads (TMR) strategy suggests that <1.2 km is a walkable distance, whereas national active transport statements suggest <2 km [24]. We transferred trips made by car of <2 km to walking and trips between 2–5 km to cycling. We assumed that trips of 5–16 km made by car can be replaced by public transport. Our assumption of replaceable distances is aligned with past data on average distance travelled reported by mode of 1 km for walking, 4 km for cycling and 15 km for public transport [25].

Shifts from car occupant (driver and passenger) to active transport trips per week reflected the age and sex distributions of transport mode share at baseline. To estimate the distance of walking and cycling trips that replaced car occupant trips, the mean kilometres travelled was calculated for each of the three distance categories (<2; 2-<5 km; 5–16 km) separately for each age and sex group (Table 2). These estimates were used to estimate the health impact of increasing PA levels. To estimate the health impact of increasing PA levels by shifting car occupant travel to public transport walking, the mean minutes of public transport walking was calculated for each of the three distance categories separately for each age and sex group. Decreases in car occupant kilometres travelled per year were used to estimate the health impact of exposure to PM_2.5_ and road trauma.

**Table 2:** Mode share travel targets.

Baseline		Travel targets		Change in number of weekday trips (% mode)		
9% walking		15% walking		291,834 (65%)		
1% cycling		5% cycling		196,864 (390%)		
8% public transport		14% public transport		291,834 (73%)		
82% car occupants		66% car occupants		-780,531 (-19%)		
**Baseline transport mode distribution by age and sex**						
	**17–49, male**	**17–49, female**	**50–74, male**	**50–74, female**	**75 +, male**	**75 +, female**
**Walking**	26%	41%	14%	15%	1%	2%
**Cycling**	61%	20%	17%	2%	0%	0%
**Public transport**	37%	40%	8%	12%	1%	2%
**Mean increase in weekday trips from baseline to the travel target scenario, by age and sex**^a^						
**Persons**	475,481	486,670	217,226	226,625	35,807	49,980
**Walking**	0.8	1.24	0.95	0.99	0.56	0.49
**Cycling**	1.27	0.4	0.77	0.10	0	0
**Public transport**	1.15	1.19	0.53	0.76	0.46	0.63
**Baseline car trip length for each distance category, by age and sex**						
**< 2km**	1.27	1.27	1.17	1.18	1.19	1.19
**2–5 km**	3.37	3.32	3.34	3.31	3.18	3.04
**5–16 km**	9.47	8.91	9.57	9.44	8.74	7.31
**Baseline mean minutes of walking to get to/from public transport, by age and sex**						
	13.39	13.15	12.58	12.77	10.03	13.31
**Decrease in car occupant km driven per year from baseline to the travel target scenario, by age and sex**^b^						
**Walking**	24,880,419	39,893,226	12,584,681	13,781,943	1,240,537	1,531,142
**Cycling**	105,524,358	33,231,580	29,150,158	3,732,041	-	-
**Public transport**	269,397,592	267,323,368	57,491,462	84,207,133	7,424,688	11,975,262

^**a**.^ Equals: Change in number of weekday trips*Baseline mode distribution by age and sex/Persons in age and sex group*5 (weekdays).

^**b**.^ Equals: Travel target scenario mean increase in daily trips by age and sex * Baseline car trip length by distance category by age and sex*Persons in age and sex group*260 (weekdays in a calendar year).

### Quantification of health outcomes and health care costs

Health outcomes and health care costs were estimated over the lifetime of the Brisbane adult population with 2013 serving as the baseline year. Health outcomes were derived from changes in average population PA, exposure to annual mean ambient fine particulate matter (PM_2.5_), exposure to on-road PM_2.5_, and road trauma (injuries and fatalities). PM_2.5_ is a widely-used proxy for exposure to air pollution during travel [4]. We used a mathematical model based on the proportional multi-cohort multi-state life table Markov model (MSLT) developed for the Assessing Cost-Effectiveness in Prevention project (ACE-prevention) [26–29].

**Fig 1:**
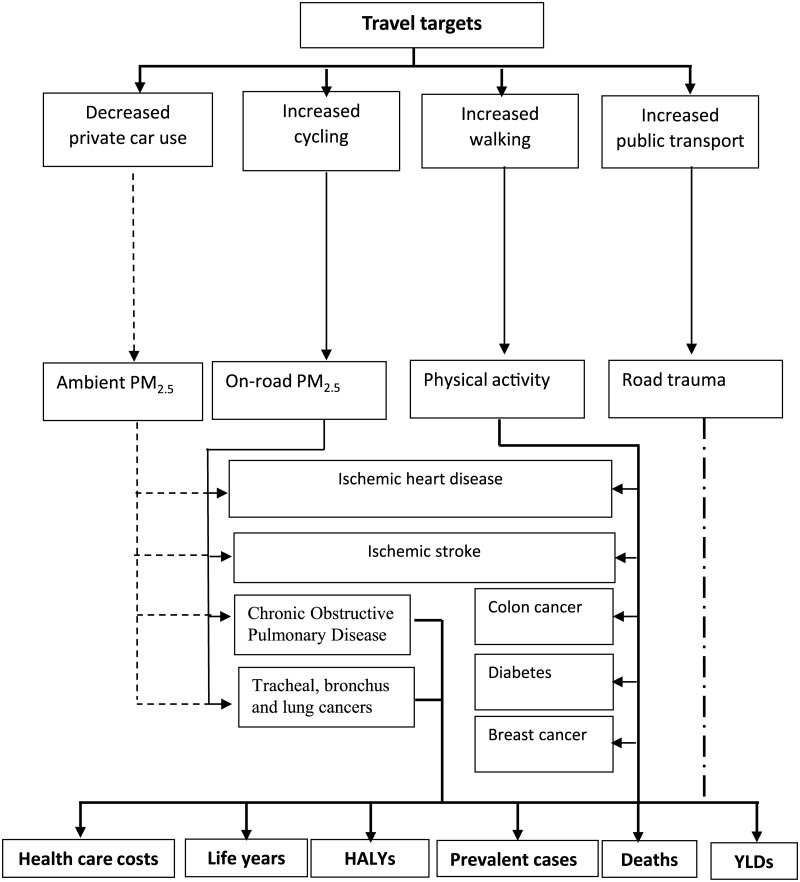
Analytical framework. Achieving the travel targets results in increased cycling, walking and use of public transport at the expense of private car travel (thick solid lines), which leads to gains in HALYs, gained life years, reduced health care costs, prevented/increased prevalent cases (diseases) and changes in death rates (thick lines at the bottom of the graph). Averted years lived with disability were estimated for road trauma. The effect of PA and PM_2.5_ were modelled via their impact on incidence of diseases (thin lines) and road trauma via its impacts on disability and mortality (captured by HALYs and YLDs) (interrupted thick lines). The effect of less private car use was quantified as improvements in ambient PM_2.5,_ which benefits the population as a whole (interrupted thin lines).

We compared health outcomes and costs for a scenario in which age- and sex-specific travel patterns persist from 2013 to 2026, with a scenario in which proposed travel targets would be achieved by 2026. Outcomes and costs associated with the travel targets scenario were assumed to be the result of a gradual increase in active travel. Outcomes were estimated by dividing status-quo and scenario populations into 5-year age groups (20–24 to 95 plus) by sex and simulating each cohort in the MSLT until everyone dies or reaches the age of 100. Health outcomes included: health-adjusted life years (HALYs), life years, prevalent cases, deaths and years lived with disability (YLDs). HALYs are estimated as years of life lived adjusted for disease-related quality of life. Health care costs of included diseases were calculated by dividing total cost of a disease by the number of incident or prevalent cases for each 5-year age-sex group. For road injuries we estimated the health care costs per year lived with disability. We present undiscounted health outcomes [30] and used a 3% annual discount rate for health care costs [31]. We tested the sensitivity of our results to discounting health at 3% and health care costs at 5% (Table N in S1 File). Ninety-five percent uncertainty intervals were determined for all outcome measures by Monte Carlo simulation (2,000 iterations), using the Excel add-in tool Ersatz (Epigear, Version 1.34) [32]. Fig 1 depicts the study’s analytical framework. Input parameters and uncertainty distributions are presented in Table 3 and S1 File.

**Table 3:** Proportional multi-state life table Markov model input parameters.

Input parameter	Uncertainty/Parameters^a^	Source
**Baseline and travel target scenario**		
2013 mortality rates and population numbers	N/A	Australian Bureau of Statistics [33, 34]
Years live with disability (YLD) (all causes and road trauma)	N/A	Institute of Health Metrics and Evaluation [35]
Incidence and case fatality modelled diseases^b^	N/A	DisMod II [32] from Global Burden of Disease (GBD) 2013 data [36] and Australian Institute of Health and Welfare (AIHW) data [37] (see S1 File ‘Notes on DisMod II modelling’)
Disability weights modelled diseases	N/A	Prevalence and years lived with disability from GBD 2013 (see S1 File ‘Disability weights’)
Relative risk, PA	Normal (Ln RR)^c^	Danaei et al. [38]
Relative risks, ischaemic heart disease and ischaemic stroke due to diabetes	Normal (Ln RR)^c^	Asia Pacific Cohort Studies Collaboration [39]
Relative risk, PM_2.5_	Normal (Ln RR)^c^	World Health Organization [40], Hamra et al. [41]
Mediating effect of diabetes in the causal pathway between PA and ischemic heart disease and ischemic stroke	Normal	GBD^d^ 2013 study (see ref [42] and page 711)
PA categories	Dirichlet	National Nutrition and Physical Activity Survey Basic Confidentialised Unit Record File (CURF) [8]
PA categories derived from MET-minutes	Lognormal	National Nutrition and Physical Activity Survey Basic Confidentialised Unit Record File (CURF) [8]
MET-minutes (walking = 3.5, cycling = 6.8, moderate PA = 5, vigorous PA = 7.5)	N/A	Ainsworth et al. [43] for walking and cycling; Australian Bureau of Statistics [8] for walking, moderate and vigorous PA
Health care costs	N/A	AIHW [44]: All diseases except COPD [45] were indexed to 2013 using AIHW-reported health sector indices [46, 47]. Denominators for calculating per case costs (incidence, prevalence and years lived with disability) [48]. See Tables L and M in S1 File.
Discount rate for health care costs	N/A	Murray et al. [30] for health; Gold et al. [31] for health care costs
**Travel targets scenario**		
Mode share distribution by age and sex	Dirichlet	South East Queensland Household Travel survey [19]
Mean distance travelled by car occupants per distance: categories by age and sex	Lognormal	South East Queensland Household Travel survey [19]
Total distance travelled by mode	N/A	South East Queensland Household Travel survey [19]
PM_2.5_ concentration	N/A	Queensland Goverment [49]
Source apportionment to motor vehicles PM_2.5_	N/A	Friend et al. [50], Environmental Protection Agency [51]
Road trauma	Gamma	Queensland Government Department of Transport and Main Roads [52] for crash data. Assumed standard deviation of 20% from the mean.

^**a**.^ Uncertainty distributions around input parameters are presented in S1 File.

^**b**.^ Breast cancer, colon cancer, tracheal, bronchus and lung cancer, type 2 diabetes, chronic obstructive pulmonary disease, ischemic heart disease and ischemic stroke.

^**c**.^ A modified version of the log of the relative risk function was used to avoid a skewed lognormal distribution [53].

^**d**.^ Global Burden of Disease (GBD).

**ABS**: Australian Bureau of Statistics; **AIHW**: Australian Institute of Health and Wellbeing; **IHME**: Institute of Health Metrics; **NNPA**: National Nutrition and Physical Activity survey; **PA**: physical activity; **TMR**: Department of Transport and Main Roads; **WHO**: World Health Organization.

#### Physical activity

The ‘relative risk shift’ method for the calculation of population impact fractions (PIF) [54] was used to estimate the incidence of physical inactivity-related diseases due to changes in PA from baseline to the travel targets scenario. The calculation of the PIFs required data for prevalence of PA and relative risks (RRs) for physical inactivity-related diseases.

We derived age- and sex-specific baseline PA prevalence estimates. From national survey data collected for PA surveillance [8], we computed mean minutes spent in the previous week walking for transport, walking for recreation, doing moderate PA (excluding walking) and doing vigorous PA. We also created a PA MET-minutes/week score by multiplying the minutes spent in each of these PA types by an assigned metabolic equivalent value (MET) from a PA compendium [43] and summing them. Following Danaei et al. [38], these scores were used to categorise participants into highly active (≥1,600 MET-minutes)/wk. and ≥1h/wk of vigorous PA), recommended-level active (600 to <1,600 MET-minutes/wk and either ≥1 h of vigorous PA/wk or ≥2.5 h of moderate PA/wk), insufficiently active <600 MET-minutes/wk or <2.5 h/wk of moderate PA) and inactive (0 MET-minutes/wk of moderate or vigorous PA).

The additional minutes walked, cycled and in public transport were summed to create an expected additional minutes in active transport per week. To estimate the increase in minutes walked, the expected increase in the number of walking trips per week was multiplied by the expected reduction in km driven by car (Table 2) and divided by walking speed (Table E in S1 File). The additional minutes cycled per week was estimated in the same way. The additional minutes per week spent in public transport walking, defined as walking to/from public transport destinations, was estimated as the product of the expected additional public transport trips in the travel targets scenario and the mean time spent walking per public transport trip at baseline (Table 2). Last, the expected additional minutes in active transport were multiplied by marginal MET-rates to derive the scenario mean energy expenditure by age and sex.

RRs were estimated for a four-tier dose-response relationship (inactive, low active, moderately active and highly active) between PA and health outcomes, as done by Danaei et al. (2009). For modelling the relationship, categorical RRs were converted into continuous functions. Assuming non-linear associations between PA and health outcomes, we fitted log-linear functions with a power transformation of mean energy expenditure per PA category (inactive, low active, moderately active and highly active) serving as the independent variable and RRs reported in the source data serving as the dependent variable (0.5 for type 2 diabetes, ischemic heart diseases and breast cancer and 0.25 for ischemic stroke and colon cancer) [38]. Next, we used the estimated parameters (intercept and slope) to estimate RRs for the baseline and travel targets scenario per PA category. Those in the highly active group had a RR of 1.00 in the source study, implying no additional benefit from extra PA [38]. Because type 2 diabetes is a risk factor for cardiovascular disease, estimated RRs incorporated the increased risk of ischemic heart disease and ischemic stroke among those with type 2 diabetes. To avoid double counting we reduced the PIFs for PA with ischemic heart disease (14%) and ischemic stroke (8%) [42].

#### PM2.5

Health effects from PM_2.5_ were estimated at two levels: (1) health effects for the population from a decrease in exposure attributable to a reduction in private car kilometres travelled and (2) health effects for the individual from an increase in exposure attributable to an increase in active travel kilometres travelled. We used information on the exposure-response relationship for PM_2.5_ and health, as well as differential exposure to PM_2.5_ between the baseline and travel targets scenario. We used RRs from a World Health Organization meta-analysis study on the long-term health effects of exposure to PM_2.5_ on cardiovascular and respiratory disease [40]. We applied the cardiovascular RR to ischemic heart disease and ischemic stroke. We also incorporated lung cancer using the RR from a meta-analysis by Hamra et al. [41]. We calculated RRs compared to baseline exposure [7] to modify incidence rates of PM_2.5_ related diseases in the MSLT.

Our calculations required data on background PM_2.5_ concentrations for the baseline and travel targets scenario. We calculated average background levels of PM_2.5_ for the Brisbane area from hourly measurements collected between 2006 to 2014 at six regulatory monitoring sites located across Brisbane, which all used standard reference methods to measure PM_2.5_ mass concentrations (i.e. tapered element oscillating microbalances) [49]. We calculated the arithmetic mean from all available data excluding sites with less than 75% of the measurements for a given year. This was to avoid seasonal bias in the estimates. We used source apportionment data specific to Brisbane to estimate the proportion of PM_2.5_ emissions attributable to motor vehicles [50]. Source apportionment data were collected from two sites in Brisbane, one urban and one suburban, with considerable variation in the proportion of PM_2.5_ attributable to motor vehicles (7% and 30%). We took the average and compared our results to other measurements at the national level, with our average of 18% comparing well with national estimates of 17% (see Table 9 in ref [55]). A range of motor vehicles contribute to traffic emissions, including passenger vehicles, but source apportionment data for Brisbane were only available for motor vehicles as a whole, rather than passenger *vs*. heavy vehicles. We used data for Queensland to allocate the proportion of motor vehicle emissions to passenger cars and buses (28% and 10%) [51]. We conducted a sensitivity analysis that assumed that passenger vehicles emit 65% of the motor vehicle-related PM_2.5_ [56]. We also tested the sensitivity of our results of using the two extreme source apportionment values.

Motor vehicle-related PM_2.5_ for the travel targets scenario was reduced in equal proportion to the reduction in passenger car kilometres travelled per year. The decrease in passenger car kilometres travelled was estimated using data from the household travel survey [19] as the total distance replaced by active modes and public transport (Table 2). The distance travelled by bus was increased proportionally to the increase in bus trips in the travel targets scenario.

To estimate the population-level effect we calculated the difference in exposure as the difference between PM_2.5_ concentrations for the baseline and travel targets scenario. Changes in individual exposure to PM_2.5_ were estimated by considering mode-specific concentrations and respiratory ventilation rates. Mode-specific exposure compared to background exposure and ventilation rates were those used in a recent study by Tainio et al. [7] (cycle = 2 and walk = 1.1). Cycling can also take place in designated paths for pedestrian and bicyclist traffic. In Brisbane it is permitted to cycle on sidewalks [57]. We tested the sensitivity of our results to cycling having the same mode specific exposure to PM_2.5_ as walking. Weekly inhaled PM_2.5_ dose was estimated by multiplying the time during a week spent sleeping, in other activities and in a passenger car (baseline) or walking/cycling/public transport for the travel targets scenario [7]. It was assumed that while not in a passenger car or walking/cycling/public transport, people are exposed to the background levels of PM_2.5._

#### Road trauma

We used crash data collected by the police and available in the Road Crash database, maintained by the Department of Transport and Main Roads, to assess road trauma [52]. We summarised the number of fatalities and road injuries (includes hospitalisation and medically treated casualties) by victim and striking mode. Our figures are for 2009, to match available travel data. We estimated a baseline rate (*R*_0_) of fatalities and injuries per kilometres travelled by victim and striking mode based on methods developed in past research [58] (Equation F in S1 File). We estimated the number of fatalities and injuries under the travel targets scenario based on the baseline rate and the kilometres travelled per mode (Equation G in S1 File). To reflect the declining risk of injuries with increasing traffic volume (commonly referred as “safety-in-numbers effect”) [59], we took the square root of kilometres travelled by victims and striking modes [60]. We tested the sensitivity of our results to the assumption of a linear association between road casualties and traffic volume.

We incorporated the health impact of road fatalities in the MSLT by multiplying baseline mode specific mortality rates (pedestrian, bicyclist, passenger car occupant and motorcyclists) by a factor reflecting the change in fatalities in the travel targets scenario (road fatalities by mode travel targets scenario/Road fatalities by mode baseline). The same approach was used for injuries; however, the impact was evaluated on mode-specific years lived with disability.

S1 File (Section 2.3) provides further information on the calculations and details on the data sources used for calculating the health effects of exposure to PA, PM_2.5_ and road trauma. Table N in S1 File presents a summary of sensitivity scenarios.

## Results

To achieve the travel targets, trips by car occupants need to decrease for all distance categories except for trips >17 km by the same percentage points as increases in active transport modes (Table 4).

**Table 4:** Percentage of trips made by distance travelled and transport mode, for baseline and travel target scenario.

Mode	<2km		2-5km		6-16km		17km+	
	Baseline	Target	Baseline	Target	Baseline	Target	Baseline	Target
Car occupant	65%	40%	90%	73%	87%	69%	84%	84%
Walking	34%	59%	4%	4%	0%	0%	0%	0%
Bicycle	1%	1%	1%	18%	1%	1%	0%	0%
Public Transport	0%	0%	5%	5%	12%	30%	16%	16%

We assumed that achieving the travel targets would require an increase in weekly walking and cycling across all age and sex categories. Table 5 depicts the change in weekly trips from private car travel to active transport by age and sex. Table 6 presents the weekly increase in minutes walked and cycled by age and sex. These estimates account for baseline travel patterns by age and sex (Table 2).

**Table 5:** Mean trips per week (weekdays only) for baseline and travel targets scenario, by age and sex.

	Car occupant		Walking		Bicycle		Public Transport		Sum
Age (years) and sex	Baseline	Target	Baseline	Target	Baseline	Target	Baseline	Target	
17–49, male	10.34	7.13	1.12	1.91	0.31	1.58	1.40	2.55	13.17
17–49, female	14.01	11.19	1.64	2.88	0.09	0.48	1.44	2.62	17.18
50–74, male	12.57	10.31	1.23	2.19	0.16	0.93	0.63	1.16	14.59
50–74, female	11.89	10.05	1.33	2.32	0.02	0.12	0.91	1.67	14.15
75 plus, male	9.05	8.04	0.71	1.27	-	-	0.45	0.90	10.21
75 plus, female	5.83	4.71	0.64	1.13	-	-	0.76	1.39	7.23

**Table 6:** Additional mean minutes per week of transport physical activity undertaken in the travel targets scenario compared to the baseline scenario (statu-quo), by age and sex.

Age (years) and sex	Additional mean minutes per week of physical activity		
	Walk for transport	Bicycle for transport	Public Transport (+ walk)^a^
17–49, male	13	16	16
17–49, female	21	4	16
50–74, male	15	10	7
50–74, female	16	2	10
75+, male	8	0	5
75+, female	8	0	8

^**a**.^ Estimated as the additional public transport trips in the travel targets scenario multiplied by the mean minutes walking in a public transport trip estimated from the household travel survey.

Road fatalities and injuries per 100 million kilometres travelled would decrease for all victim modes affected by the travel targets scenario, except for car occupants, for whom the fatality and injury rates would be almost identical to those at baseline (Table 7). Assuming a linear association between kilometres travelled and road trauma (that is, removing the ‘safety in numbers’ effect) resulted in a less significant reduction in rates (sensitivity scenario).

**Table 7:** Road trauma rates per 100 million kilometres travelled by transport mode.

	Baseline		Base case^a^		Sensitivity scenario^b^	
	Fatalities	Injuries	Fatalities	Injuries	Fatalities	Injuries
**Pedestrian**	1.43	125.59	1.06	90.67	1.44	119.95
**Cyclist**	2.24	165.04	1.46	106.21	2.18	156.48
**Car occupant**	0.09	23.67	0.10	24.37	0.09	23.12
**Motorcyclist**	3.91	153.38	3.84	149.23	3.77	145.28

^**a**.^ Non-linear association kilometres travelled and road trauma.

^**b**.^ Linear association kilometres travelled and road trauma.

Background PM_2.5_ would decrease marginally in the travel targets scenario, with variations depending on the attribution of PM_2.5_ to motor vehicles and passenger cars (Table 8).

**Table 8:** PM_2.5_ values baseline and sensitivity scenarios.

	Baseline	Base case^a^	Sensitivity scenarios		
			Low level apportionment MV^b^	High level apportionment MV^c^	Passenger cars 65% MV emissions^d^
**PM**_**2.5**_ **(μm/m**^**3**^**)**	6.964	6.957	6.962	6.940	6.920
**Change emissions from passenger cars (%)**		-0.41%	-0.15%	-0.66%	-0.94%
**Change emissions from buses (%)**		0.31%	0.12%	0.31%	0.31%
**Total effect (%)**		-0.10%	-0.04%	-0.35%	-0.63%

^**a**.^17% of PM2.5 attributable to motor vehicles (MV). Of MV emission, 28% corresponds to passenger cars and 10% to buses.

^**b**.^7% of PM2.5 attributable to MV. Of MV emission, 28% corresponds to passenger cars and 10% to buses.

^**c**.^30% of PM2.5 attributable to MV. Of MV emission, 28% corresponds to passenger cars and 10% to buses.

^**d**.^ 17% of PM2.5 attributable to MV. Of MV emission, 65% corresponds to passenger cars and 10% to buses.

### Health and health care cost outcomes

**Fig 2:**
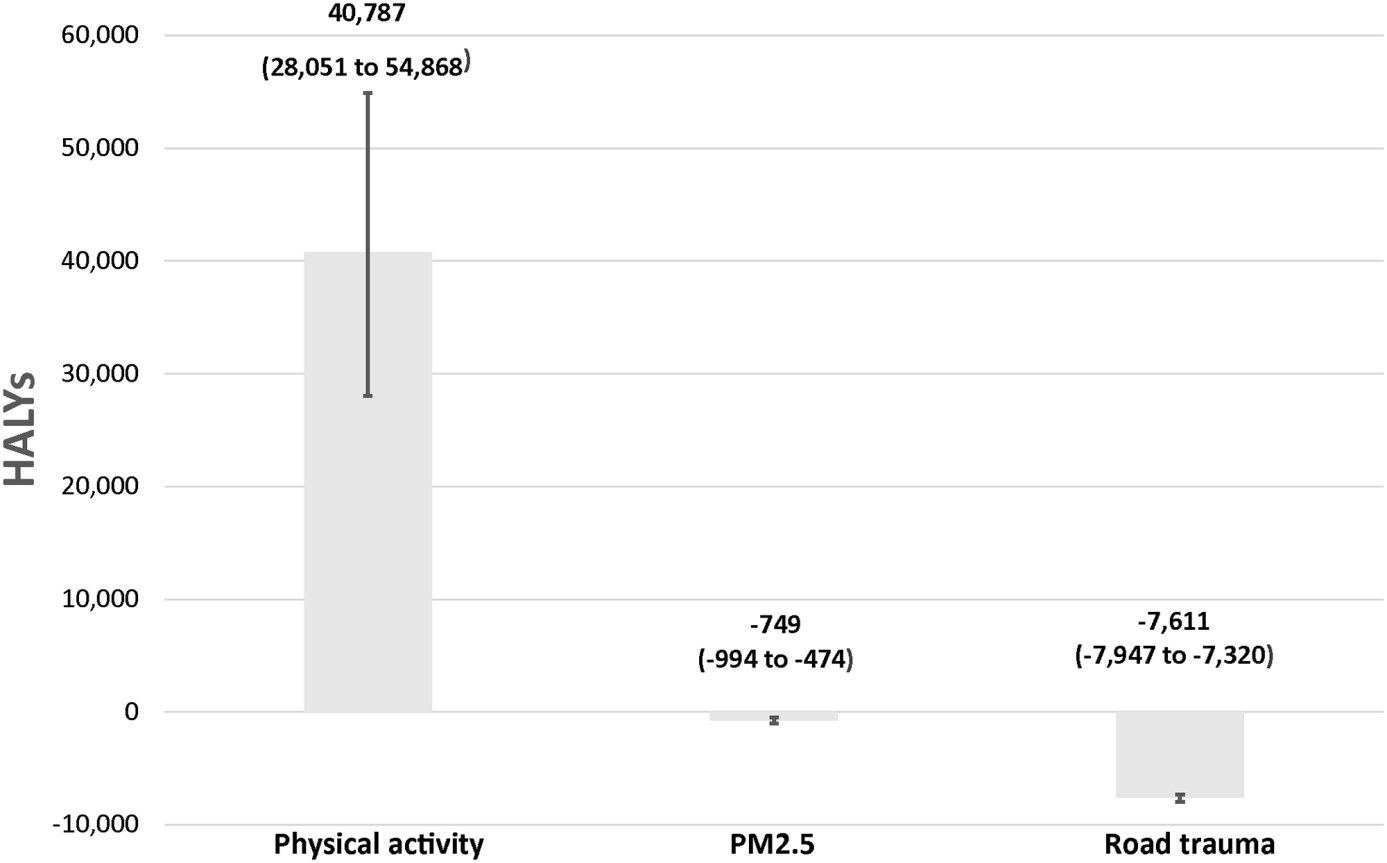
HALYs by risk factor over the life course of the Brisbane adult population (95% uncertainty interval).

**Fig 3:**
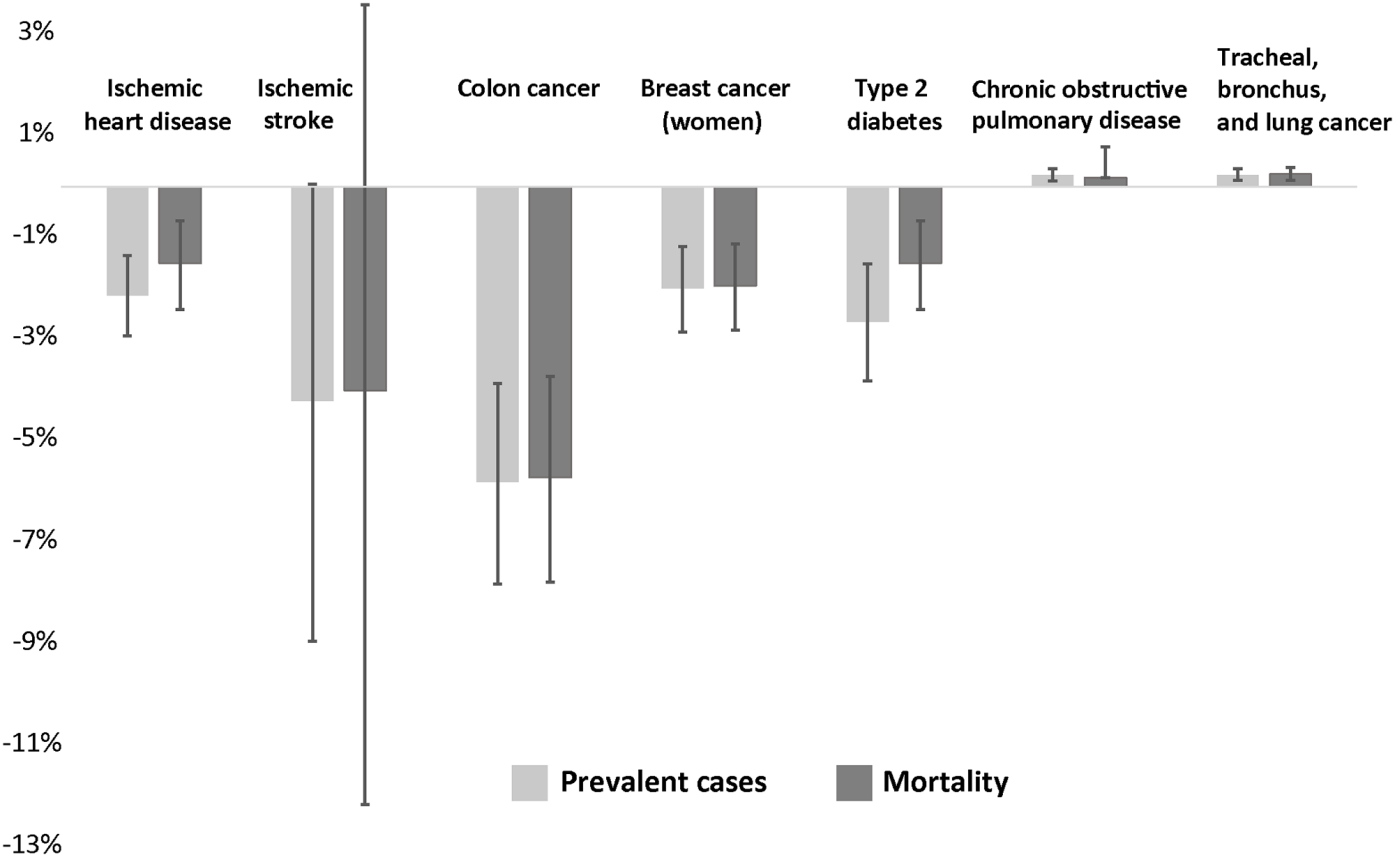
Percent change in disease prevalence and mortality over the life course of the Brisbane adult population (error bars indicate the 95% uncertainty interval).

Over the life course of the Brisbane adult population in 2013 (860,000 persons), slightly over 32,000 HALYs and 28,000 life years could be gained if the proposed travel targets were achieved by 2026 (Table 9). We estimated that significant savings ($AU312 million) in health care costs could be accrued in the travel targets scenario. However, an increase in the number of life years lived would translate into additional health care costs of $AU129 million. Most of the health gains would result from improvements in population levels of PA, with exposure to PM_2.5_ and road trauma having small negative impacts (Fig 2). The number of prevalent cases decreased for all modelled diseases except for respiratory diseases (Fig 3 and Table 10). We estimated a reduction in mortality from ischemic heart disease, colon cancer, breast cancer and type 2 diabetes. The uncertainty interval for the reduction for ischemic stroke mortality includes 0. This can be explained by the weak association between PA and ischemic stroke and the effect of additional cases from added life years and exposure to PM_2.5_. Our results suggest that road trauma may lead to a 30% (95% UI 29% to 32%) increase in mortality (593 deaths) and a 6.6% (95% UI 6.2% to 7.0%) increase in the number of years lived with disability (3,339) over the life course of the Brisbane adult population. The results are most sensitive to the discount factor applied to health outcomes and the dose-response relationship between road trauma and kilometres travelled by mode. A higher discount rate for health care costs and health outcomes implies a lower present value. Assuming a linear association between road trauma and kilometres travelled, there would be greater negative effects compared to the base case. S1 File section 4 provides further results from the sensitivity analysis, including for intermediate outcomes.

**Table 9:** Health care costs and health outcomes for base case by sex over the life course of the Brisbane adult population (95% uncertainty interval).

	Health-adjusted life years (thousand)	Life years (thousand)	Health care costs total (millions)^a^	Other health care costs in added LYs total (millions)
**Total**	32.6 (19.6 to 46.8)	28.1 (13.1 to 44.0)	-$312 (-$463 to -$173)	$129 ($49 to $213)
**Females**	17.6 (9.2 to 26.3)	16.2 (6.2 to 26.8)	-$139 (-$221 to -$63)	$80 ($22 to $141)
**Males**	15.0 (9.8 to 20.9)	11.9 (6.8 to 17.8)	-$173 (-$246 to -$107)	$49 ($26 to $76)

^***a***.^ Negative values are savings.

**Table 10:** Change in prevalent cases and mortality over the life course of the Brisbane adult population (95% uncertainty interval).

Disease	Prevalent cases	Mortality
Ischemic heart disease	-44,902 (-61,765 to -28,463)	-1,416 (-2,275 to -624)
Ischemic stroke	-14,343 (-30,420 to 182)	-1,504 (-4,558 to 1,342)
Colon cancer	-19,630 (-26,409 to -13,091)	-406 (-552 to -265)
Breast cancer (women)	-13,184 (-18,815 to -7,763)	-158 (-228 to -091)
Type 2 diabetes	-90,440 (-130,002 to -51,905)	-325 (-474 to -169)
Chronic obstructive pulmonary disease	7,831 (3,881 to 12,026)	130 (049 to 217)
Tracheal, bronchus and lung cancer	356 (192 to 531)	81 (42 to 122)

## Discussion

Significant health gains could be made if government targets for reductions in private car travel and increases in active transport were achieved in Brisbane, Australia. In this study we estimated the effect of achieving active transport targets by 2026 (5% cycling, 15% walking and 14% public transport). A significant increase in active transport translates into substantial improvements to population levels of PA, health gains and health care costs savings. Health benefits from increases in PA are significantly higher than the potential negative effects of increases in air pollution and road trauma exposure. Our results were the most sensitive to the choice of discount rate and the shape of the dose-response curve for road trauma and kilometres travelled.

These results are consistent with those from the limited number of previous studies, which were conducted in the United States, Spain, Brazil, England and Australia [58, 60–63]. Similar to our findings, the findings from those studies indicate that the greatest contributions to health gains from replacing passenger cars kilometres travelled to active transport were due to improvements in PA. However, it is difficult to make a direct comparison among studies because of differences in the scenarios analysed, methods applied and the high context specificity of the research. For example, Maizlish and colleagues [60] modelled the potential health impact of replacing short car trips with walking and cycling and introducing low-emission cars in the San Francisco Bay Area in California. The study findings indicate that over 5,000 disability-adjusted life years per million people annually could be averted by replacing motorised travel with active modes and the introduction of low–emission driving. To our knowledge, there is only one previous Australian study that assessed the health outcomes of PA, air pollution and road trauma from a shift towards sustainable alternative transport [61]. Projecting travel patterns in 2030 for the City of Adelaide, the authors found that replacing 40% of motor vehicle kilometres travelled with cycling (10%) and public transport (30%) would translate into over 7,500 averted DALYs annually for a projected population of 1.4 million people (5,357 per million people). We calculated annual values and we found gains of 1,700 HALYs per million people. The studies by Maizlish et al. and Xia et al. used a prediction modelling framework based on the comparative risk assessment approach (CRA) [30]. As we explain in the following section, CRA tends to overestimate the change in burden of disease.

### Strengths and limitations

To our knowledge, this is the first study to quantify the potential health and health care cost outcomes of a shift towards active transport using the well-established method of the proportional multi-state life table Markov model (MSLT). The MSLT allows for the long-term estimation of health and economic outcomes [64]. By using this approach, we were able to incorporate a gradual shift from private car trips to active modes. Also, the interaction between multiple diseases is partly accounted for, with proportions of the population being able to be in more than one disease state [64]. This avoids over-estimating outcomes as a result of summing health outcomes attributable to each disease individually, though it does not fully account for the clustering of diseases in a subset of individuals. Another source of overestimation in past studies arises from the use of the CRA approach based on Global Burden of Disease (GBD) estimates [6]. GBD studies estimate DALYs as the sum of years of life lost (YLL) and years lost due to disability (YLD) [30]. The mortality rates used in GBD studies to estimate YLL are for a hypothetical population that has the lowest observed mortality at every age [30], whereas we used the current mortality rates for the population in question for the life years lived component. On the other hand, since we did not assume a trend towards lower mortality rates in future years, our results may be overly cautious. Furthermore, in GBD studies, YLLs are not adjusted for disability; hence, their use in estimating intervention effects results in over-estimation, which our life table approach avoids. Another way of seeing this is that estimated changes in morbidity using CRA methods do not allow for the impact of life expectancy increases on morbidity [65]. While the changes in deaths and prevalence using the MSLT are in some ways more accurate than those from a CRA approach, it should be noted that that the average age of death and incident disease will change, and thus, the disease burden will be, on average, shifted to later in life. Thus, changes in HALYs offer a more appropriate measure of gain than changes in life course prevalence. Past studies using the CRA approach also found that health gains were about twice as large when predicting the impact of PA on all-cause mortality compared to on disease specific mortality [58].

Limitations should be highlighted. Our estimates of shifts from private cars to alternative travel modes in the intervention scenario are compared to a scenario in which travel patterns by age and sex remain constant over time, and we assume that each group will become more active in proportion to its current activity level. This assumption could be challenged: when cycling becomes more common, it also tends to become more equitable [66], and providing safe and direct cycling routes may encourage more women and older people to commute by bicycle [67]. More sophisticated methods based on propensity analysis accounting for trip distance and hilliness, as previously done for cycling in England, could further refine our active travel estimations [68]. There is also a risk of bias in our baseline travel estimates as the data collection periods excluded summer months when people may be less likely to use active commuting due to the heat. While we used the best available road trauma data for Brisbane, our use of an overall road trauma risk is a limitation. Past studies indicate differential road trauma risk by age and sex [69] and also by road type [60]. Because we only used data from Brisbane, the number of events in some categories was small and may be a result of chance rather than accurately reflecting risk. Also under-reporting of injuries is common in police data [70, 71]. Another limitation arises from using self-reported data for physical activity and travel patterns. Our application of the MSLT assumes that a proportion of the population that is sufficiently active (≥1,600 MET-minutes)/wk. and ≥1h/wk. of vigorous PA) receives no benefit from additional PA, which may be an underestimation of health impacts [72]. Another source of underestimation arises from an incomplete inclusion of diseases. There is growing evidence suggesting a causal association between PA, lung cancer, endometrial cancer and dementia [73, 74]. A limitation of this and similar studies [75] results from not knowing the exact shape of the dose-response curve (e.g. linear, curvilinear, log-normal) for the relationship between PA and health. Recent studies that have used a continuous exposure to assess the association between PA and health indicate the greatest benefits at low levels of PA [72, 76]. Another consideration is that the level of ambient air pollution in Brisbane is one of the lowest observed in the world [77], and relative risks for PM_2.5_ are based on data from more polluted locations, resulting in uncertainty at the lowest part of the exposure-response curve [78]. We incorporated the effect of trends in incidence and case fatality in our model and assumed that all other model parameters would remain constant. Variations in these parameters could influence results upward or downward. Migration and natural population growth were not considered, although our use of a life table approach resulted in the effects of population aging being included. The strong population growth that would be expected for Brisbane would act to increase the health gains forecasted in this paper.

### Implications

Increasing active transport can help reduce the health and economic burden of low levels of PA observed in Australia. However, investments in infrastructure and programs to encourage behavioural change are vital to increase population uptake of active travel. For Brisbane, a long-term infrastructure plan explicitly addresses public transport as one of the priority investment strategies [79]. The plan aims to provide transit access within a 15-minute walk to 90% of the population with reasonably frequent services (15 minutes to main activity centres and 30 minutes to other destinations). In addition, the plans calls for infrastructure projects that target walking and cycling, as well as programs that support the uptake of active transport (e.g. cycle safe workshops). An update of the plan is scheduled for 2017, which will allow for the progress of the proposed investments to be assessed.

Important context-specific variables could negatively impact the uptake of active travel in Brisbane. For instance, the city sprawls widely, with an estimated population density of approximately 19 inhabitants per hectare, compared to 246 in Barcelona and 84 in Amsterdam [80]. In Barcelona, approximately 40% of the mode share is walking while in Amsterdam, where cycling is a popular mode of transport, 22% of the mode share is cycling [81]. Hence, efforts by transport department to increase active transport should be supported by land use planning policies that result in more compact living, to facilitate the provision of public transport and access to destinations within walking/cycling distance [82]. Local plans for Brisbane for the next 25 years aim to increase population density [83].

While transport and land use planning are moving towards compact living and accessible transport in Brisbane, some opposition could arise from the community. In Sydney (Australia’s largest city), local businesses indicated opposition to new cycling infrastructure due to concerns over its potential negative impact on commerce, mostly from reduced parking space [84]. However, after construction parking issues were hardly mentioned in interviews with business owners, and new business owners indicated that the new cycleway had a positive influence on their decision to move to the area. Increasing population density has been widely opposed by local resident groups in Australian cities [85]. However, recent research in Canada showed that negative attitudes towards compact living could be substantially diminished by imparting positive messages of the potential public benefits (e.g. improvement in air quality, reduction of traffic congestion, improvements in walkability) of such developments [86].

### Conclusion

Shifting towards active transport is a long-term process that needs investment and continuity in governments’ strategic planning. In Queensland an updated regional plan was released early in 2017; however, no specific transport targets were set [83]. The current research indicates that continuing working towards achieving the proposed mode shares of 5% cycling, 15% walking and 14% public transport would deliver considerable health and economic gains. From a societal perspective, all would benefit from improved quality of life and savings in health care costs.

Even though our results are highly context specific, they support the international evidence of the health benefits of investing in active transport.

## Supporting information

S1 File (DOCX)

## References

[1] Global report of urban health: equitable, healthier cities for sustainable development [Internet]. 2016. http://www.who.int/kobe_centre/measuring/urban-global-report/ugr_full_report.pdf?ua=1.

[2] Sallis JF, Bull F, Burdett R, Frank LD, Griffiths P, Giles-Corti B, et al Use of science to guide city planning policy and practice: how to achieve healthy and sustainable future cities. The Lancet. 2016;388(10062):2936–47. 10.1016/S0140-6736(16)30068-X 27671670

[3] Brown V, Zapata-Diomedi B, Moodie M, Veerman JL, Carter R. A systematic review of economic analyses of active transport interventions that include physical activity benefits. Transport Policy. 2016;45:190–208. 10.1016/j.tranpol.2015.10.003.

[4] Mueller N, Rojas-Rueda D, Cole-Hunter T, de Nazelle A, Dons E, Gerike R, et al Health impact assessment of active transportation: a systematic review. Prev Med. 2015;76:103–14. 10.1016/j.ypmed.2015.04.010. 25900805

[5] De Nazelle A, Nieuwenhuijsen MJ, Antó JM, Brauer M, Briggs D, Braun-Fahrlander C, et al Improving health through policies that promote active travel: a review of evidence to support integrated health impact assessment. Environ Int. 2011;37(4):766–77. 10.1016/j.envint.2011.02.003 21419493

[6] Stevenson M, Thompson J, de Sá TH, Ewing R, Mohan D, McClure R, et al Land use, transport, and population health: estimating the health benefits of compact cities. The Lancet. 2016;388(10062):2925–35. 10.1016/S0140-6736(16)30067-8 27671671 PMC5349496

[7] Tainio M, de Nazelle AJ, Götschi T, Kahlmeier S, Rojas-Rueda D, Nieuwenhuijsen MJ, et al Can air pollution negate the health benefits of cycling and walking? Preventive Medicine. 2016;87:233–6. 10.1016/j.ypmed.2016.02.002. 27156248 PMC4893018

[8] Australian Health Survey: Physical Activity, 2011–12 [Internet]. 2015 [cited 22 September 2015]. http://www.abs.gov.au/ausstats/abs@.nsf/Lookup/D4495467B7F7EB01CA257BAC0015F593?opendocument.

[9] GBD Compare Data Visualization [Internet]. 2016 [cited 19 January 2017]. http://vizhub.healthdata.org/gbd-compare.

[10] Environmental Issues: Waste Management, Transport and Motor Vehicle Usage [Internet]. 2012 [cited 15 August 2016]. http://www.abs.gov.au/ausstats/abs@.nsf/mf/4602.0.55.002.

[11] Connecting SEQ 2031 –An Integrated Regional Transport Plan for South East Queensland [Internet]. 2011. http://www.ppt.asn.au/pubdocs/connecting_seq2031+(1).pdf.

[12] Queensland Cycle Strategy 2011–2021 [Internet]. 2015 [cited 2 March 2016]. http://www.tmr.qld.gov.au/Travel-and-transport/Cycling/Strategy.aspx.

[13] Brisbane Active Transport Strategy 2012–2026 [Internet]. 2012 [cited 15 February 2015]. https://www.brisbane.qld.gov.au/sites/default/files/active_transport_strategy_2012-2026.pdf.

[14] Australia: the healthiest country by 2020-National Preventative Health Strategy [Internet]. Commonwealth of Australia. 2009 [cited 5 November 2016]. http://www.preventativehealth.org.au/internet/preventativehealth/publishing.nsf/Content/CCD7323311E358BECA2575FD000859E1/$File/nphs-roadmap.pdf.

[15] Australian Local Government Association, Bus Industry Confederation, Cycling Promotion Fund, National Heart Foundation of Australia, International Association of Public Transport. An Australian vision for active transport 2010 [cited 2014 15 November]. http://www.heartfoundation.org.au/SiteCollectionDocuments/Active-Vision-for-Active-Transport-Report.pdf.

[16] Brisbane suburbs [Internet]. 2015 [cited 25 February 2016]. http://www.brisbane.qld.gov.au/about-council/council-information-rates/brisbane-suburbs.

[17] Brisbane Community Profiles [Internet]. 2016 [cited 25 February 2016]. http://www.brisbane.qld.gov.au/about-council/governance-strategy/business-brisbane/business-opportunities/brisbane-community-profiles.

[18] Population by Age and Sex, Regions of Australia [Internet]. 2014 [cited 10 April 2016]. http://www.abs.gov.au/AUSSTATS/abs@.nsf/DetailsPage/3235.02013?OpenDocument.

[19] 2009 South East Queensland Household Travel Survey [Internet]. Queensland Goverment,. 2009 [cited 15 December 2015]. https://data.qld.gov.au/dataset/2009-south-east-queensland-household-travel-survey.

[20] Department of Transport and Main Roads (TMR). South-East Queensland Travel Survey 2009-Survey Procedures and Documentation. 2010;V2.2. Unpublished.

[21] 2009–12 South East Queensland Household Travel Survey [Internet]. 2016 [cited 15 November 2016]. https://data.qld.gov.au/en/dataset/2009-12-south-east-queensland-household-travel-survey/resource/2086c5cd-966e-49e2-ad53-f2a9d789cbab.

[22] 2901.0—Census Dictionary, 2006 (Reissue) [Internet]. 2006 [cited 22 December 2016]. http://www.abs.gov.au/ausstats/abs@.nsf/0/413876F3BAE9CC70CA25720A000C428B?opendocument.

[23] Australia's commuting distance: cities and regions [Internet]. Bureau of Infrastructure, Transport and Regional Economics,. 2015 [cited 16 October 2016]. https://bitre.gov.au/publications/2015/files/is_073.pdf.

[24] Walking, Riding and Access to Public Transport-Suporting Active Travel in Australian Communities [Internet]. 2013. https://infrastructure.gov.au/infrastructure/pab/active_transport/files/infra1874_mcu_active_travel_report_final.pdf.

[25] Queensland Household Travel Survey summary reports [Internet]. Queensland Goverment. 2016 [cited 16 December 2016]. http://www.tmr.qld.gov.au/Community-and-environment/Research-and-education/Queensland-Household-Travel-Survey-summary-reports.aspx.

[26] Barendregt JJ, Van Oortmarssen GJ, Van Hout BA, Van Den Bosch JM. Coping with multiple morbidity in a life table. Math Popul Stud. 1998;7(1):29–49. 10.1080/08898489809525445 12321476

[27] Barendregt JJ, Oortmarssen vGJ, Murray CJ, Vos T. A generic model for the assessment of disease epidemiology: the computational basis of DisMod II. Popul Health Metr. 2003;1(1):4-. 10.1186/1478-7954-1-4 12773212 PMC156029

[28] Cobiac LJ, Vos T, Barendregt JJ. Cost-effectiveness of interventions to promote physical activity: a modelling study. Plos Med. 2009;6(7):e1000110–e. 10.1371/journal.pmed.1000110 . 19597537 PMC2700960

[29] Vos T, Carter R, Barendregt JJ, C. M, Veerman J, Magnus A, et al. Assessing Cost-Effectiveness in Prevention (ACE-Prevention): Final Report. University of Queensland, Brisbane and Deakin University, Melbourne: 2010.

[30] Murray CJL, Ezzati M, Flaxman AD, Lim S, Lozano R, Michaud C, et al GBD 2010: design, definitions, and metrics. The Lancet. 2012;380(9859):2063–6. 10.1016/S0140-6736(12)61899-6 23245602

[31] Gold MR. Cost-effectiveness in health and medicine. New York: Oxford University Press; 1996.

[32] Barendregt JJ. EpiGear International 2012 [cited 2015 1 Mar]. http://www.epigear.com/index_files/prevent.html.

[33] Deaths, Australia, 2015 [Internet]. Australia Bureau of Statistics. 2016 [cited 15 October 2016]. http://www.abs.gov.au/ausstats/abs@.nsf/mf/3302.0.

[34] Estimated Resident Population By Single Year of Age Australia [Internet]. 2016 [cited 10 October 2016]. http://www.abs.gov.au/AUSSTATS/abs@.nsf/DetailsPage/3101.0Dec%202015?OpenDocument.

[35] GBD Compare [Internet]. IHME, University of Washington. 2015 [cited 15 July 2016]. https://vizhub.healthdata.org/gbd-compare/.

[36] Global Burden of Disease (GBD) [Internet]. 2015 [cited 25 July 2016]. http://www.healthdata.org/gbd.

[37] Australian Cancer Incidence and Mortality (ACIM) books [Internet]. 2016 [cited 4 August 2016]. http://www.aihw.gov.au/acim-books.

[38] Danaei G, Ding EL, Mozaffarian D, Taylor B, Rehm J, Murray CJL, et al The preventable causes of death in the United States: comparative risk assessment of dietary, lifestyle, and metabolic risk factors. PLoS Med. 2009;6(4):e1000058 10.1371/journal.pmed.1000058 19399161 PMC2667673

[39] Asia Pacific Cohort Studies Collaboration. The Effects of Diabetes on the Risks of Major Cardiovascular Diseases and Death in the Asia-Pacific Region. Diabetes Care. 2003;26(2):360–6. 10.2337/diacare.26.2.360 12547863

[40] World Health Organization. WHO Expert Meeting: Methods and tools for assessing the health risks of air pollution at local, national and international level. Copenhagen: WHO Regional Office for Europe, 2014.

[41] Hamra GB, Guha N, Cohen A, Laden F, Raaschou-Nielsen O, Samet J, et al Outdoor particulate matter exposure and lung cancer: a systematic review and meta-analysis. Environ Health Perspect. 2014;122:906–11. 10.1289/ehp.1408092 24911630 PMC4154221

[42] GBD 2013 Risk Factors Collaborators. Global, regional, and national comparative risk assessment of 79 behavioural, environmental and occupational, and metabolic risks or clusters of risks in 188 countries, 1990–2013: A systematic analysis for the Global Burden of Disease Study 2013. The Lancet. 2015;386(10010):2287–323. 10.1016/S0140-6736(15)00128-2 26364544 PMC4685753

[43] Ainsworth BE, Haskell WL, Herrmann SD, Meckes N, Bassett DR Jr., Tudor-Locke C, et al 2011 compendium of physical activities: a second update of codes and MET values. Med Sci Sports Exerc. 2011;43(8):1575–81. Epub 2011/06/18. 10.1249/MSS.0b013e31821ece12 . 21681120

[44] Australian Institute of Health and Welfare. Disease costs and impact study data. Australian Institute of Health and Welfare, 2001.

[45] How much is spent on COPD? [Internet]. 2016 [cited 12 October 2016]. http://www.aihw.gov.au/copd/expenditure/.

[46] Australian Institute of Health and Welfare. Health expenditure Australia 2009–2010. Canberra: 2011 Health and welfare expenditure series no. 46. Cat. no. HWE 55.

[47] Australian Institute of Health and Welfare. Health expenditure Australia 2013–14. Canberra: 2015 Cat. no. HWE 63.

[48] Global Burden of Disease Study 2015 (GBD 2015) Results [Internet]. 2016 [cited 19 October 2016]. http://ghdx.healthdata.org/gbd-results-tool.

[49] Air Quality Monitoring [Internet]. Queensland Goverment,. 2015 [cited 10 August 2016]. https://data.qld.gov.au/dataset?q=air+quality.

[50] Friend AJ, Ayoko GA, Stelcer E, Cohen D. Source apportionment of PM at two receptor sites in Brisbane, Australia. Environmental Chemistry. 2011;8(6):569–80. 10.1071/EN11056

[51] Air Emissions Inventory-South East Queensland Region [Internet]. Queensland Goverment,. 2004. http://s3.amazonaws.com/zanran_storage/www.epa.qld.gov.au/ContentPages/15521293.pdf.

[52] Crash data from Queensland roads [Internet]. Queensland Goverment 2014 [cited 1 September 2016]. https://data.qld.gov.au/dataset/crash-data-from-queensland-roads.

[53] Barendregt JJ. The effect size in uncertainty analysis. Value in health: the journal of the International Society for Pharmacoeconomics and Outcomes Research. 2010;13(4):388–91. Epub 2010/07/28. 10.1111/j.1524-4733.2009.00686.x . 20659273

[54] Barendregt JJ, Veerman JL. Categorical versus continuous risk factors and the calculation of potential impact fractions. J Epidemiol Community Health. 2010;64(3):209–12. 10.1136/jech.2009.090274 19692711

[55] Australian Motor Vehicle Emission Inventory for the National Pollutant Inventory (NPI) [Internet]. 2014. http://www.npi.gov.au/resource/australian-motor-vehicle-emission-inventory-national-pollutant-inventory-npi.

[56] Kristensson A, Johansson C, Westerholm R, Swietlicki E, Gidhagen L, Wideqvist U, et al Real-world traffic emission factors of gases and particles measured in a road tunnel in Stockholm, Sweden. Atmospheric Environment. 2004;38(5):657–73. 10.1016/j.atmosenv.2003.10.030.

[57] Bicycle road rules and safety [Internet]. 2016 [cited 28 September 2016]. https://www.qld.gov.au/transport/safety/rules/wheeled-devices/bicycle/#riding.

[58] Woodcock J, Givoni M, Morgan AS. Health impact modelling of active travel visions for England and Wales using an Integrated Transport and Health Impact Modelling Tool (ITHIM). PLoS One. 2013;8(1):e51462 10.1371/journal.pone.0051462 23326315 PMC3541403

[59] Elvik R, Bjørnskau T. Safety-in-numbers: A systematic review and meta-analysis of evidence. Safety Science. 2017;92:274–82. 10.1016/j.ssci.2015.07.017.

[60] Maizlish N, Woodcock J, Co S, Ostro B, Fanai A, Fairley D. Health Cobenefits and Transportation-Related Reductions in Greenhouse Gas Emissions in the San Francisco Bay Area. American Journal of Public Health. 2013;103(4):703–9. 10.2105/AJPH.2012.300939 23409903 PMC3673232

[61] Xia T, Nitschke M, Zhang Y, Shah P, Crabb S, Hansen A. Traffic-related air pollution and health co-benefits of alternative transport in Adelaide, South Australia. Environ Int. 2015;74:281–90. 10.1016/j.envint.2014.10.004 25454245

[62] Rojas-Rueda D, de Nazelle A, Teixidó O, Nieuwenhuijsen MJ. Replacing car trips by increasing bike and public transport in the greater Barcelona metropolitan area: A health impact assessment study. Environment International. 2012;49:100–9. 10.1016/j.envint.2012.08.009. 23000780

[63] Sá TH, Duran AC, Tainio M, Monteiro CA, Woodcock J. Cycling in São Paulo, Brazil (1997–2012): Correlates, time trends and health consequences. Preventive Medicine Reports. 2016;4:540–5. 10.1016/j.pmedr.2016.10.001. 27761356 PMC5067980

[64] Briggs ADM, Wolstenholme J, Blakely T, Scarborough P. Choosing an epidemiological model structure for the economic evaluation of non-communicable disease public health interventions. Population Health Metrics. 2016;14(1):17 10.1186/s12963-016-0085-1 27152092 PMC4857239

[65] Mytton O, Tainio M, Ogilvie D, Panter J, Cobiac LJ, Woodcock J. Impact of increases in physical activity on disease burden, considering the effect of increasing life expectancy and reduced incidence of disease: a multi-state lifetable modelling study. Eur J Epidemiol. 2017;32(3):235–50. 10.1007/s10654-017-0235-1. 28258521 PMC5380706

[66] Aldred R, Woodcock J, Goodman A. Does More Cycling Mean More Diversity in Cycling? Transport Reviews. 2015;36(1):28–44. 10.1080/01441647.2015.1014451

[67] Aldred R, Elliott B, Woodcock J, Goodman A. Cycling provision separated from motor traffic: a systematic review exploring whether stated preferences vary by gender and age. Transport Reviews. 2016;37(1):29–55. 10.1080/01441647.2016.1200156 28190905 PMC5259802

[68] Lovelace R, Goodman A, Aldred R, Berkoff N, Abbas A, Woodcock J. The Propensity to Cycle Tool: An open source online system for sustainable transport planning. 2016 10.5198/jtlu.2016.862

[69] Santamariña-Rubio E, Pérez K, Olabarria M, Novoa AM. Gender differences in road traffic injury rate using time travelled as a measure of exposure. Accident Analysis and Prevention. 2014;65:1–7. 10.1016/j.aap.2013.11.015 24384384

[70] Yannis G. Modeling road accident injury under-reporting in Europe. European transport research review. 2014;6(4):425–38. 10.1007/s12544-014-0142-4

[71] Watson A, Watson B, Vallmuur K. Estimating under-reporting of road crash injuries to police using multiple linked data collections. Accident Analysis & Prevention. 2015;83:18–25. 10.1016/j.aap.2015.06.011. 26162640

[72] Kyu HH, Bachman VF, Alexander LT, Mumford JE, Afshin A, Estep K, et al Physical activity and risk of breast cancer, colon cancer, diabetes, ischemic heart disease, and ischemic stroke events: systematic review and dose-response meta-analysis for the Global Burden of Disease Study 2013. BMJ. 2016;354 10.1136/bmj.i3857 27510511 PMC4979358

[73] Blondell SJ, Hammersley-Mather R, Veerman JL. Does physical activity prevent cognitive decline and dementia?: A systematic review and meta-analysis of longitudinal studies. BMC Public Health. 2014;14(1):510. 24885250 10.1186/1471-2458-14-510 PMC4064273

[74] Hamer M, Chida Y. Physical activity and risk of neurodegenerative disease: a systematic review of prospective evidence. Psychol Med. 2009;39(1):3–11. Epub 2008/06/24. 10.1017/S0033291708003681 . 18570697

[75] Woodcock J, Edwards P, Tonne C, Armstrong BG, Ashiru O, Banister D, et al Public health benefits of strategies to reduce greenhouse-gas emissions: urban land transport. The Lancet. 2009;374(9705):1930–43. 10.1016/S0140-6736(09)61714-1 19942277

[76] Smith AD, Crippa A, Woodcock J, Brage S. Physical activity and incident type 2 diabetes mellitus: a systematic review and dose–response meta-analysis of prospective cohort studies. Diabetologia. 2016:1–19. 27747395 10.1007/s00125-016-4079-0 PMC6207340

[77] World Health Organization. Global Ambient Air Pollution 2016 [cited 2016 18 Oct]. http://maps.who.int/airpollution/.

[78] Burnett RT, Pope CA, Ezzati M, Olives C, Lim SS, Mehta S, et al An integrated risk function for estimating the Global Burden of Disease attributable to ambient fine particulate matter exposure. Environ Health Perspect. 2014;122(4):397–403. 10.1289/ehp.1307049 24518036 PMC3984213

[79] Brisbane Long Term Infrastructure Plan 2012–2031 [Internet]. Brisbane City Council,. 2012. http://www.brisbane.qld.gov.au/sites/default/files/Brisbane_Long_Term_Infrastructure_Plan-full.pdf.

[80] Comparing the densities of Australian, European, Canadian, and New Zealand cities [Internet]. 2016. https://chartingtransport.com/2015/11/26/comparing-the-densities-of-australian-and-european-cities/.

[81] TEMS—The EPOMM Modal Split Tool [Internet]. N/D. http://www.epomm.eu/tems/result_cities.phtml?more=1.

[82] Giles-Corti B, Foster S, Ryan K. Increasing density in Australia: maximising the health benefits and minimising harm. Melbourne: National Heart Foundation of Australia, 2012.

[83] Shaping SEQ-Draft South East Queensland Regional Plan [Internet]. Queensland Goverment. 2016. http://www.dilgp.qld.gov.au/noindex/shapingseq/draft-south-east-queensland-regional-plan.pdf.

[84] Crane M, Rissel C, Greaves S, Standen C, Ming Wen L. Neighbourhood expectations and engagement with new cycling infrastructure in Sydney, Australia: Findings from a mixed method before-and-after study. Journal of Transport & Health. 2016;3(1):48–60. 10.1016/j.jth.2015.10.003.

[85] Cook N, Taylor E, Hurley J. At home with strategic planning: reconciling resident attachments to home with policies of residential densification. Australian Planner. 2013;50(2):130–7. 10.1080/07293682.2013.776982

[86] Doberstein C, Hickey R, Li E. Nudging NIMBY: Do positive messages regarding the benefits of increased housing density influence resident stated housing development preferences? Land Use Policy. 2016;54:276–89. 10.1016/j.landusepol.2016.02.025.

