# Supplementary material for: A shift from motorised travel to active transport: What are the potential health gains for an Australian city?
Source: PLoS One. 2017 Oct 11;12(10):e0184799. doi: 10.1371/journal.pone.0184799 (PMC5636090; doi:10.1371/journal.pone.0184799)
Supplement: S1 File — (DOCX) [file pone.0184799.s001.docx]

**Supplementary material**

**A shift from motorised travel to active transport: What are the potential health gains for an Australian city?**

**Contents**

[List of tables i](#_Toc492539103)

[List of figure ii](#_Toc492539104)

[1 Introduction 1](#_Toc492539105)

[2 Proportional multi-state multi-cohort life table Markov model 2](#_Toc492539106)

[2.1 Changes in disease frequency 2](#_Toc492539107)

[2.2 Data sources 3](#_Toc492539108)

[2.2.1 Breast cancer, colon cancer and tracheal, bronchus and lung cancer 5](#_Toc492539109)

[2.2.2 Disability weights 7](#_Toc492539110)

[2.3 Included risk factors 7](#_Toc492539111)

[2.3.1 Physical activity 7](#_Toc492539112)

[2.3.2 PM_2.5_ 12](#_Toc492539113)

[2.3.3 Road trauma 14](#_Toc492539114)

[2.4 Health care costs and costs in added life years 16](#_Toc492539115)

[3 Sensitivity analysis 17](#_Toc492539116)

[4 Additional results to the main manuscript 18](#_Toc492539117)

[5 Validation tests 20](#_Toc492539118)

# List of tables

[Table A DisMod II input data and procedures for modelled diseases 4](#_Toc492539119)

[Table B Mean (SE) MET-minutes/wk. per physical activity category 9](#_Toc492539120)

[Table C Relative risks (SE log (RR)) of disease at different levels of physical activity 9](#_Toc492539121)

[Table D Relative risks comparison example 10](#_Toc492539122)

[Table E Active transport speed 12](#_Toc492539123)

[Table F Level of data completeness (%) by monitoring site and estimated annual mean for background PM_2.5_ (µg/m^3^) 13](#_Toc492539124)

[Table G Relative Risks for exposure to PM_2.5_ 13](#_Toc492539125)

[Table H Parameters and calculations for the change in exposure at the individual level for the travel targets scenario 14](#_Toc492539126)

[Table I Baseline road fatalities 15](#_Toc492539127)

[Table J Baseline road injuries 15](#_Toc492539128)

[Table K Person and vehicle kilometres travelled per capita for baseline and travel targets scenario 16](#_Toc492539129)

[Table L Disease cost per case 16](#_Toc492539130)

[Table M Costs for all other diseases in added life years 17](#_Toc492539131)

[Table N Univariate sensitivity analysis parameters 17](#_Toc492539132)

[Table O Health care costs and health outcomes for sensitivity scenarios (95% uncertainty interval) 18](#_Toc492539133)

[Table P Population impact fraction for PA-related diseases for travel targets scenario compared to baseline by age and sex used to modify incidence rates in MSLT^a^ 19](#_Toc492539134)

[Table Q Relative risks for PM_2.5_-related diseases for travel targets scenario compared to baseline by age and sex used to modify incidence rates in MSLT 20](#_Toc492539135)

[Table R Model tests^a^ 21](#_Toc492539136)

# List of figure

[Figure A Figure Schematic description of a proportional MSLT 2](#_Toc492539137)

[Figure B Conceptual disease model 3](#_Toc492539138)

[Figure C Breast cancer incidence and prevalence for females 5](#_Toc492539139)

[Figure D Colon cancer prevalence and incidence females and males 6](#_Toc492539140)

[Figure E Tracheal, bronchus and lung cancer 6](#_Toc492539141)

[Figure F Prevalence physical activity 8](#_Toc492539142)

[Figure G Relative Risks fitting function example 11](#_Toc492539143)

#

# Introduction

In this document we expand on the methods description and results in the main manuscript. Section two explains the methods and input parameters for the multi-cohort proportional multi-state life table Markov Model (MSLT). In section three we present results for the sensitivity analyses. Section four expands on results not presented in the main manuscript. Lastly, we present the results of validity tests performed.

# Proportional multi-state multi-cohort life table Markov model

A schematic description of the proportional multi-state life table is presented below (Figure A) only for the travel targets scenario population in the model (as it is derived from the baseline population).

r_i_ Tracheal, bronchus and lung cancer cancers

m

x

-

m

x

+

q

x

l

x

L

x

e

x

w

x

-

w

x

+

Lw

x

HALE

x

i

x

p

x

m

x

w

Disease process 1

Disease process 2

i

x

p

x

m

x

w

Risk factor

m

x

-

m

x

+

q

x

l

x

L

x

e

x

w

x

-

w

x

+

Lw

x

HALE

x

i

x

p

x

m

x

w

Life table

Disease process 1

Disease process 2

i

x

p

x

m

x

w

m_i_ Tracheal, bronchus and lung cancer cancers

Risk factor

Figure A Figure Schematic description of a proportional MSLT

This figure describes the interaction between life-table parameters and disease parameters. All the parameters are age specific denoted with x, i is incidence, p is prevalence and m is mortality, w is disability adjustment, q is probability of dying, l is number of survivors, L is life years, Lw is disability adjusted life years and HALE is health adjusted life expectancy, ‘-‘denotes parameter related to diseases or causes that specifically excludes modelled diseases or injuries and ‘+‘ relates to all modelled diseases in the model. A change in the determinant of health (physical activity and PM_2.5_) translates into changes in incidence (i_x_), which changes disease specific prevalence (p_x_) and mortality (m_x_). For presentation purposes we only depict two diseases processes, however, in this study we modelled 7 diseases (ischemic heart disease, ischemic stroke, breast cancer, colon cancer, type 2 diabetes, COPD and tracheal, bronchus and lung cancer). Road fatalities (m_i_) impact directly on mortality and road injuries (r_i_) impact on years lived with disability, which are captured in Lw in the schematic description.

## Changes in disease frequency

The seven included diseases are modelled applying a set of differential equations to describe the transition between four states (healthy, diseased, dead from the disease and dead from all other causes) (Barendregt et al., 2003) (Figure B). Transition probabilities among the four states are based on rates of incidence, remission, case fatality and background mortality. A change in exposure to the risk factors of interest (physical activity and PM_2.5_) modifies incidence via the potential impact fraction (PIF) (Equation A) or relative risk (RR) (Equation D) (Figure B). To simplify the process, remission is set to zero.

Case fatality

Healthy

Diseased

Dead

(disease)

Dead

(other)

Mortality (other)

Remission

Incidence

Mortality (other)

$PIF$/RR

$\Delta$ Exposure to risk factor

(PA and PM_2.5_)

Δ Incidence

Figure B Conceptual disease model

The model was used for each of the physical activity and PM_2.5_ related diseases. The disease conceptual model, applied to each disease separately, has four health states (healthy, diseased, dead from the disease and dead from other causes) and transition hazards between health states (Barendregt et al., 2003). PIF calculations for PA and RR for PM_2.5_ related diseases are explained in section 2.3. The diseases conceptual model is that of a multi-state life table model (box in figure).

## Data sources

Data from the Global Burden of Disease 2013 (GBD 2013) (Institute for Health Metrics and Evaluation (IHME), 2015b), Australian Institute of Health and Welfare (AIHW) (2016a) and Australian Bureau of Statistics (ABS) (2016a, 2016b) were used to populate the MSLT model. We used DisMod II to enforce internal consistency in the epidemiological estimates, and to derive parameters not provided in the data sources (Barendregt, 2012). For the MSLT model we needed estimates for incidence and case fatality rates for diseases causally associated with exposure to low levels of physical activity (breast cancer, colon cancer, type 2 diabetes, ischemic heart disease and ischemic stroke) and fine particles with a diameter of 2.5 μm or less (PM_2.5_) (chronic obstructive pulmonary disease, tracheal, bronchus and lung cancer, ischemic heart diseases and ischemic stroke). The conceptual model of DisMod II is that of a multi-state life table (Figure B): “Healthy people, defined as people unaffected by the disease being modelled, are subject to an incidence hazard, and may become diseased. When diseased they are subject to a hazard of dying from the disease, the case fatality, and to a hazard of recovery from the disease, called remission. Both healthy and diseased people are subject to the same mortality hazard from all other causes” (Barendregt et al., 2003, p. 2 ). DisMod II generates age and sex specific and internally consistent estimates for the disease modelled based on a set of differential equations. Population and mortality data were obtained from the GBD 2013 and the Australian Bureau of Statistics (see Table A). At least three of the following input parameters are required for DisMod II to estimate required input values: incidence, prevalence, remission, case fatality, duration, mortality and relative risks on all-cause mortality (for the modelled disease). Cancers were modelled with incidence and mortality from the AIHW. Remission was set to zero. For the rest of the diseases (ischemic heart disease, ischemic stroke, type 2 diabetes and COPD) we used prevalence and mortality from GBD 2013 and assumed a remission hazard of zero (same as in GBD 2013). Prevalence and disease specific mortality figures were obtained from the GBD 2013. Disease specific mortality from the GBD 2013 study are publicly available from the IHME webpage (2015a), whereas prevalence data was requested from IHME. The AIHW provides data (numbers and rates) for incidence and mortality for cancers for the year 2012 (2016a). In Table A we provide all data sources used for DisMod II as well as expand on the procedures used to generate the data. In the following section we provide more detail on the methods used for cancers.

Table A DisMod II input data and procedures for modelled diseases

| **Disease** | **Collection input parameters** | **Dataset input parameters** | **ICD-10 codes** | **Procedures^a^** |
| --- | --- | --- | --- | --- |
| Breast Cancer (females) | **Population**: ABS (2016b)  **Mortality rates**: ABS (2016a) | **Incidence and Mortality:** (AIHW 2016a) | **AIHW:** C50 | Remission set to exact zero. Manual adjustment to incidence in older ages. Moving average smoothing to incidence. Higher weight to incidence and mortality. |
| Colon cancer | **Population**: ABS (2016b)  **Mortality rates**: ABS (2016a) | **Incidence and Mortality:** (AIHW 2016a) | **AIHW:** C18 | Cubic spline interpolation for incidence and mortality (inputs) for females and cubic spline interpolation for incidence for males. Higher weight to incidence and mortality. |
| Tracheal, bronchus and lung cancer cancers | **Population**: ABS (2016b)  **Mortality rates**: ABS (2016a) | **Incidence and Mortality:** (AIHW 2016a) | **AIHW:** C33-C34 | Fitted a sigmoid curve to incidence and interpolated mortality using cubic spline. Higher weight to mortality. |
| Chronic obstructive pulmonary disease | **Population and mortality:** GBD 2013**  (IHME 2015b) | **Prevalence and disease specific mortality:** GBD 2013 (IHME 2015b) | **GBD 2013:** J40-J44.9, J47-J47.9 | Moving averages smoothing to prevalence and mortality (input). Fitted a sigmoid curve to incidence (output). Higher weight to prevalence and mortality. |
| Type 2 Diabetes | **Population and mortality:** GBD 2013 (IHME 2015b) | **Prevalence and disease specific mortality:** GBD 2013 (IHME 2015b) | **GBD 2013:** E10-E10.11, E10.3-E11.1, E11.3-E12.1, E12.3-E13.11, E13.3-E14.1, E14.3-E14.9, P70.0-P70.2, R73-R73.9, Z13.1, Z83.3 | Manually adjusted prevalence for older ages (80+) as there was an inconsistent drop. Fitted a sigmoid curve to prevalence data after manual adjustment for females. For males manual adjustment of prevalence at older ages and smoothing of curve with moving averages |
| Ischemic heart disease | **Population and mortality:** GBD 2013 (IHME 2015b) | **Prevalence and disease specific mortality:** GBD 2013 (IHME 2015b) | **GBD 2013:** I20, I21, I22, I23, I24, I25 | Cubic spline interpolation to prevalence and mortality. Fitted a sigmoid curve to case fatality (output). |
| Ischemic stroke | **Population and mortality:** GBD 2013 (IHME 2015b) | **Prevalence and disease specific mortality:** GBD 2013 (IHME 2015b) | **GBD 2013:** G45, G46.8, I63-I63.9, I64.0, I66.9, I67.2, I67.3, I67.5, I67.6, I69.3-I69.398 | Cubic spline fitting to prevalence and mortality. Higher weight to prevalence and mortality. |

a. These procedures are based on options given by DisMod II.

### Breast cancer, colon cancer and tracheal, bronchus and lung cancer

Input parameters from the AIHW for incidence and mortality were used. This decision was made because the data for prevalence from the GBD 2013 study accounted for prevalent cases for up to 10 years after becoming an incident case plus cases with long life sequelae (Supplementary Material for GBD 2013 Risk Factors Collaborators, 2015).The GBD definition of prevalence is different from that in our model, where we assume no remission, and ‘prevalence’ therefore includes all cases that ever experienced an incidence event in prior years. To avoid underestimating we used Australian data from AIHW for incidence and mortality.

In the following graphs we present outputs for the modelled cancers, comparing prevalence and incidence obtained with AIHW (incidence and mortality) and GBD 2013 (prevalence and mortality). These graphs show that prevalence generated only with GBD data is considerable lower than that based on AIHW data. Under estimation of prevalence forces DisMod II to generate lower estimates for incidence (for most ages). Furthermore, estimates generated with GBD 2013 data result in unrealistic drops of incidence in older ages followed by steep increases.


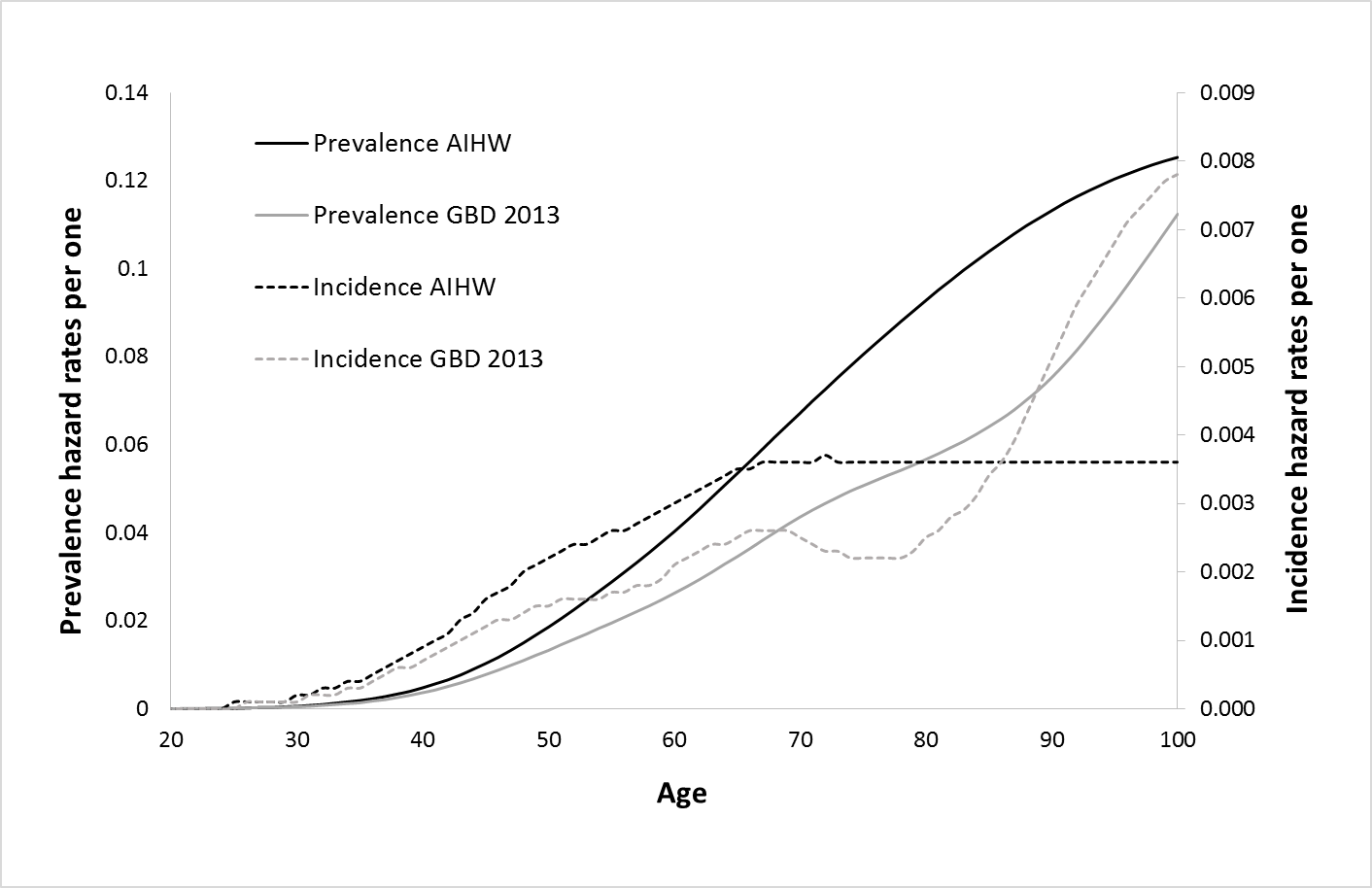


Figure C Breast cancer incidence and prevalence for females

Prevalence on the left vertical axis, incidence rates on the right.


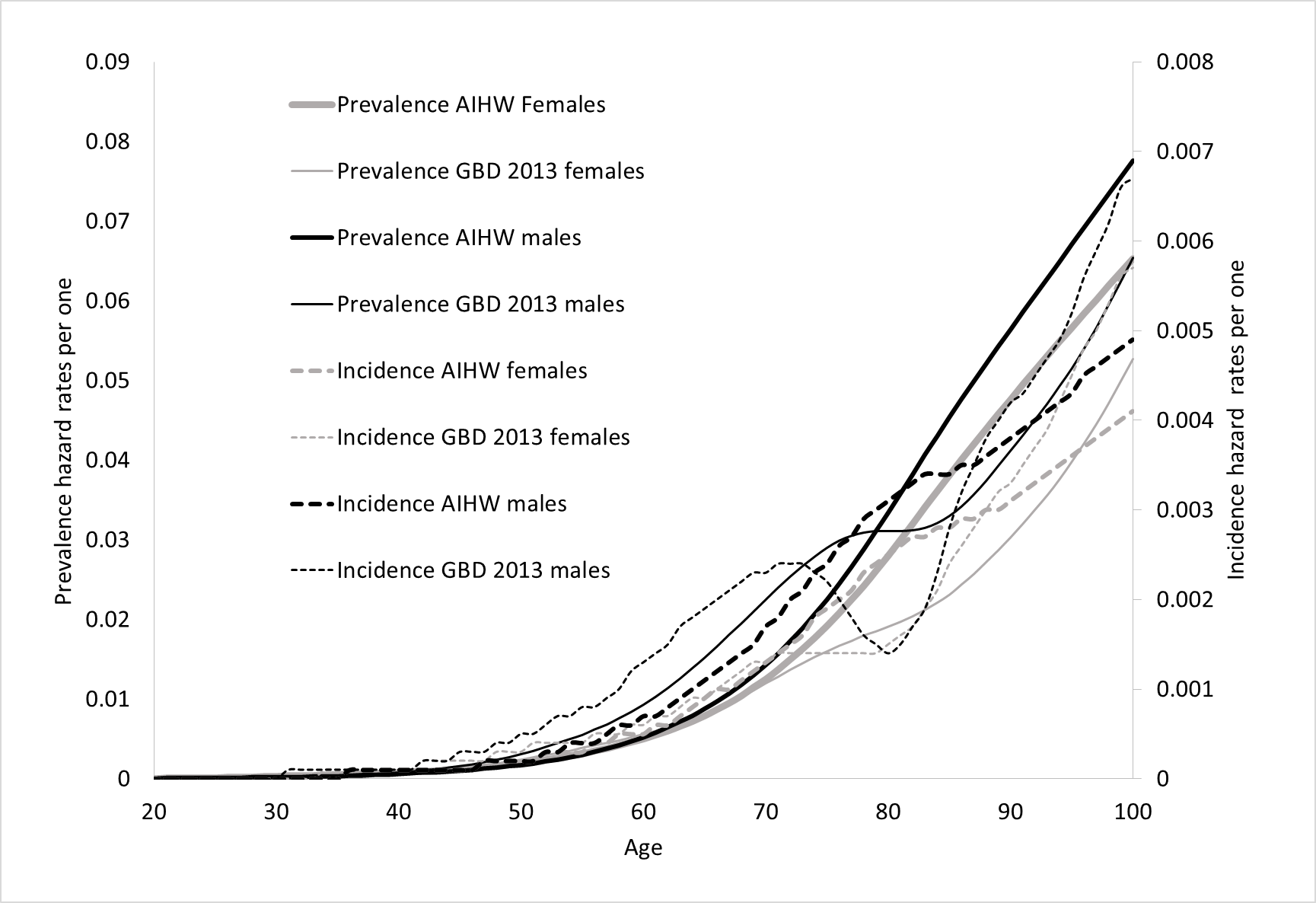


Figure D Colon cancer prevalence and incidence females and males

Prevalence on the left vertical axis, incidence rates on the right. GBD 2013 data is for Colon and rectum cancer.


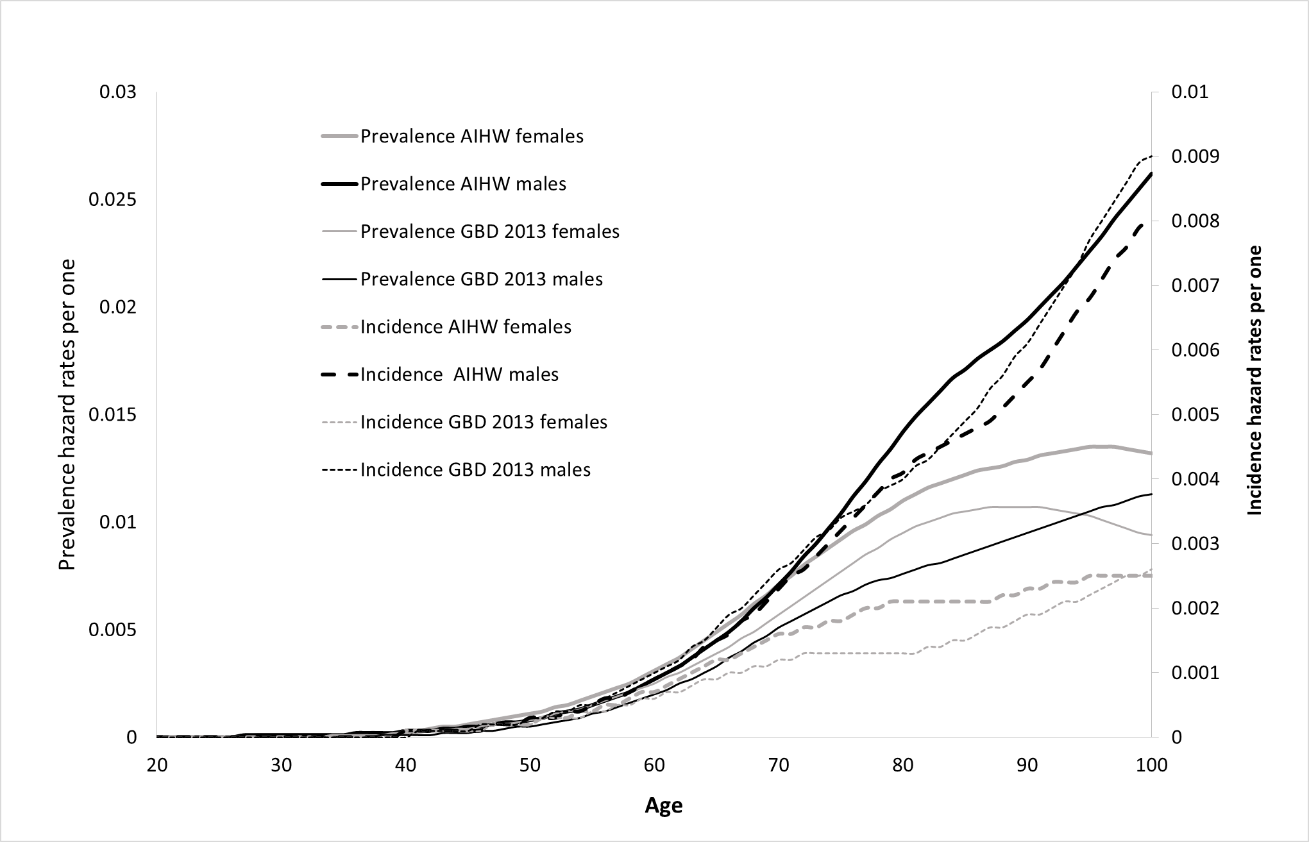


Figure E Tracheal, bronchus and lung cancer

Prevalence on the left vertical axis, incidence rates on the right.

### Disability weights

We derived disability weights (DW) from disease specific years lived with disability (YLD) and disease specific prevalence by age group (5 years) and sex. Data for YLDs were obtained from the online tool GBD Compare (IHME 2015a) and prevalence was requested from the IHME. Our calculations are based on the GBD methods for estimating YLDs as the sum of sequelae prevalence multiplied by sequelae disability weights (GBD 2015). For this study we had data at the cause level (e.g. ischemic heart disease) instead of squelae level (e.g. myocardial infarction, angina and heart failure). An age and sex specific-correction was introduced to couteract the effects of accumulating comorbid illnesses in the older age groups.

DW adjusted for total YLDs = (YLDd/Pd)/(1-YLDt)

Where YLDd is the YLD mean number per age and sex for a given disease, Pd is the prevalence (as reported in GBD 2013) for a given disease by age and sex and YLDt is total YLD rate per age and sex. For the modelled cancers we further adjust the calculated DWs by the ratio of our estimated prevalence and GBD reported prevalence. This adjustment is due to the potential over estimation of disease specific YLDs in our model given our higher estimate for prevalence. Hence in the base situation, our model reproduces the YLD burden estimated by the GBD study.

## Included risk factors

### Physical activity

We estimated the prevalence of physical activity for the Brisbane adult population (aged >20 years) from a representative sample of Australian adults. The data were collected by the Australian Bureau of Statistics and published as the Basic-CURF National Nutrition and Physical Activity Survey 2011-2012 (NNPAS) (Australian Bureau of Statistics, 2015). A sample of 9,435 adults was available to derive physical activity prevalence estimates.

Survey respondents were asked about the time they spent doing physical activity in the last week, including walking for transport, walking for recreation, moderate physical activity (excludes walking) and vigorous physical activity. A walking session was only to be recorded if the duration of the session was at least 10 minutes.

We computed Metabolic Equivalent of Tasks minutes per week (MET-mins/wk.) as minutes per week spent in each of the above mentioned activities multiplied by MET values used in the NNPAS (Australian Bureau of Statistics, 2015) (walking min x 3.5, moderate min x 5 and vigorous min x 7.5 (Ainsworth et al., 2011)). A MET represents the ratio of the work metabolic rate to the resting metabolic rate (Ainsworth et al., 2011). For each observation in the dataset we derived a measure of total MET-mins/wk. and grouped observations as per the categories by Danei et al. (2009): highly active (≥1,600 MET-minutes)/wk. and 1h/wk of vigorous PA), recommended level active (600≤MET-minutes/wk. ≤1,600 and 1 h of vigorous PA/wk. or 2.5 h of moderate PA/wk.), insufficiently active (0<MET-minutes/wk. ≤600 or <2.5 h/wk of moderate PA) and inactive (no moderate or vigorous PA). Population weighted prevalence estimates by age and sex are presented in Figures F.


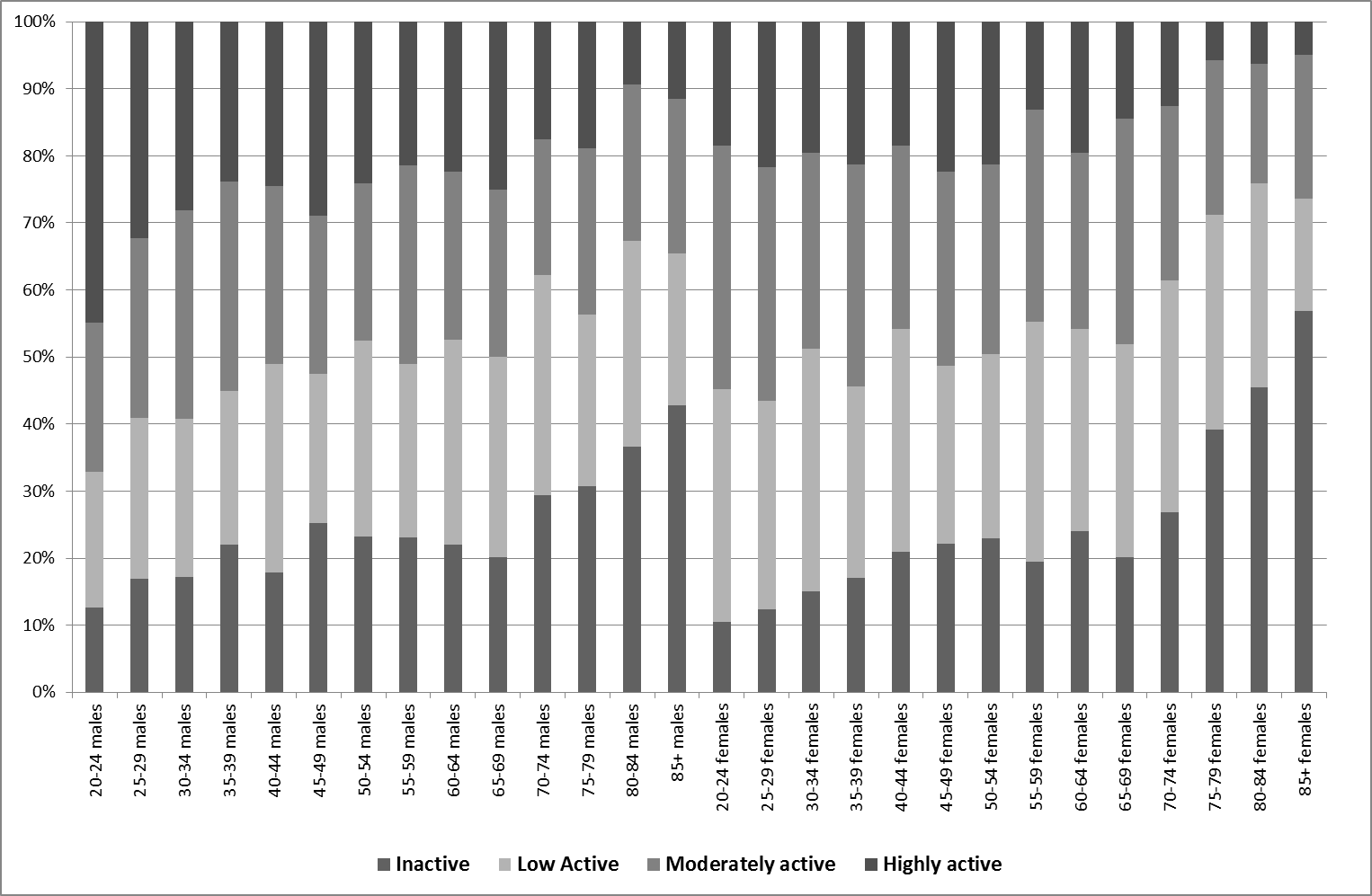


Figure F Prevalence physical activity

Mean energy expenditure in MET-minutes per week was also derived from the NNPAS and used to fit relative risk (RR) functions for the included physical activity-related diseases (Table B). Relative risks used in this study for the association physical activity-health outcomes are presented in Table C. As discussed in the main manuscript we used the “Relative Risk Shift” methods to modify incidence of physical activity diseases (Equation A).

$$PIF=\frac{\sum_{i=1}^{n} p_{i}{RR}_{i}-\sum_{i=1}^{n} p_{i}{RR}_{i}^{'}}{\sum_{i=1}^{n} p_{i}{RR}_{i}}$$

Equation A Relative risk shift

The ‘relative risk shift’ method for the calculation of the PIF (Barendregt & Veerman, 2010) was used to estimate new levels of incidence due to changes in physical activity, where $p_{i}$ is physical activity prevalence at level *i* (4 levels in this research), ${RR}_{i}$ is the relative risk of physical activity for each of the diseases associated with PA level *i* and ${RR}_{i}^{'}$ is the relative risk of physical activity for each disease after the intervention.

Table B Mean (SE) MET-minutes/wk. per physical activity category

| **Physical Activity category**^a^ | **Mean (SE)** |  |
| --- | --- | --- |
| Inactive | 0 (0) |  |
| Insufficiently active | 281.7 (4.3) |  |
| Recommended level active | 1024.7 (7.2) |  |
| Highly active | 3100.4 (57) |  |

a. Highly active (≥1,600 MET-minutes)/wk. and ≥1h/wk of vigorous PA), recommended level active (600 to <1,600 MET-minutes/wk. and either ≥1 h of vigorous PA/wk. or ≥2.5 h of moderate PA/wk.), insufficiently active <600 MET-minutes/wk. or <2.5 h/wk of moderate PA) and inactive (0 MET-minutes/wk of moderate or vigorous PA).

Table C Relative risks (SE log (RR)) of disease at different levels of physical activity

| **Outcome**  **(mortality)** | |  | **Inactive** | **Insufficiently Active** | **Recommended Level Active** | **Highly Active** |
| --- | --- | --- | --- | --- | --- | --- |
| Ischaemic heart disease^a^ | 15-69 | 1.97 (0.12) | | 1.66 (0.19) | 1.15 (0.05) | 1 |
|  | 70-79 | 1.73 (0.12) | | 1.51 (0.21) | 1.15 (0.07) | 1 |
|  | 80+ | 1.50 (0.14) | | 1.38 (0.24) | 1.15 (0.10) | 1 |
| Ischaemic stroke^a^ | 15-69 | 1.72 (0.23) | | 1.23 (0.56) | 1.12 (0.30) | 1 |
|  | 70-79 | 1.55 (0.24) | | 1.21 (0.62) | 1.12 (0.37) | 1 |
|  | 80+ | 1.39 (0.28) | | 1.18 (0.83) | 1.12 (0.59) | 1 |
| Type 2 diabetes | 15-69 | 1.76 (0.10) | | 1.50 (0.26) | 1.21 (0.12) | 1 |
|  | 70-79 | 1.60 (0.11) | | 1.43 (0.30) | 1.21 (0.16) | 1 |
|  | 80+ | 1.45 (0.14) | | 1.34 (0.39) | 1.21 (0.25) | 1 |
| Breast cancer | 15-44 | 1.56 (0.09) | | 1.41 (0.26) | 1.25 (0.17) | 1 |
|  | 45-69 | 1.67 (0.08) | | 1.41 (0.26) | 1.25 (0.17) | 1 |
|  | 70-79 | 1.56 (0.08) | | 1.36 (0.33) | 1.25 (0.23) | 1 |
|  | 80+ | 1.45 (0.11) | | 1.32 (0.45) | 1.25 (0.36) | 1 |
| Colon Cancer | 15-69 | 1.80 (0.11) | | 1.27 (0.20) | 1.07 (0.06) | 1 |
|  | 70-79 | 1.59 (0.11) | | 1.21 (0.21) | 1.07 (0.08) | 1 |
|  | 80+ | 1.39 (0.11) | | 1.16 (0.26) | 1.07 (0.12) | 1 |

**a.** Relative risks of ischaemic heart disease and ischaemic stroke due to diabetes are 2.19 (1.81-2.66) and 2.64 (1.78-3.92) respectively (Asia Pacific Cohort Studies Collaboration, 2003).

N.B. Values shown are the mean and SE

In past research (Cobiac et al., 2009), a linear assumption was made for the association of physical activity with health outcomes. However, we assumed a curvilinear association with the greatest gains at low levels of physical activity. In this section we demonstrate the more realistic fit of a log-linear curve to the association of physical activity with health outcomes. Figure G shows the estimated relative risks with the log-linear and linear functions for the baseline and travel targets scenario as well as the prevalence of physical activity per category. This example is for males in the age group 60-69 for ischemic heart disease. We added the relative risks as reported in the literature for reference and also estimates assuming a linear association. As can be observed, estimated RRs with the log–linear function of a power transformation of MET-minutes per week corresponds better to the RRs reported in the literature. The RRs for the intervention show that for the linear assumption, risk decreases equally in all categories with increasing PA (by approx. 2%), except for the highly active group which receives no benefits (Table D). In contrast, using the log-linear dose response function results in greater reduction of risk at lower levels of PA (approx. 13% for inactive group, 4% for insufficiently active and 2% for recommended level active).

Table D Relative risks comparison example

| **60-69 males** | **Inactive** | **Insufficiently Active** | **Recommended Level Active** | **Highly Active** |
| --- | --- | --- | --- | --- |
| **RR IHD log-linear baseline** | 1.95 | 1.57 | 1.29 | 1.00 |
| **RR IHD log-linear intervention** | 1.70 | 1.51 | 1.26 | 1.00 |
| **Change in risk** | **-13%** | **-4%** | **-2%** | **0%** |
| **RR IHD linear baseline** | 1.95 | 1.87 | 1.64 | 1.00 |
| **RR IHD linear intervention** | 1.92 | 1.83 | 1.61 | 1.00 |
| **Change in risk** | **-2%** | **-2%** | **-2%** | **0%** |
| **Observed (Danaei et al. 2009)** | 1.97 | 1.66 | 1.15 | 1 |

**
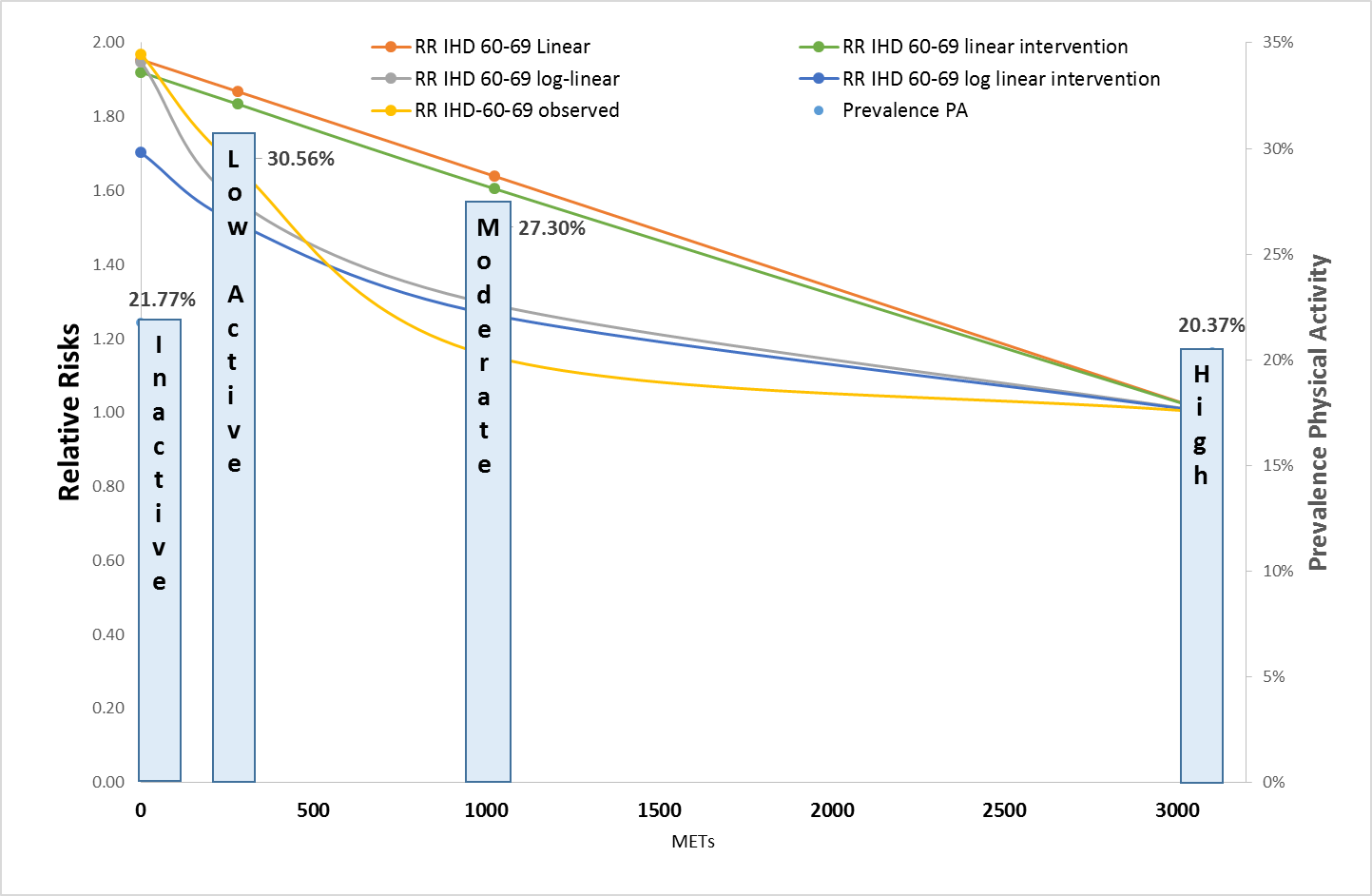
**

Figure G Relative Risks fitting function example

#### Travel targets incremental physical activity

Additional walking and cycling minutes per week (Table 6 in the main manuscript) were estimated by age and sex using data from the South East Queensland Travel survey (TMR 2009), as follows:

$$\boldsymbol{\Delta walking mins.}\boldsymbol{=}\frac{\boldsymbol{\Delta walking trips per day*Mean distance trip*5}}{\boldsymbol{Speed per hour*60}}$$

Equation B Additional minutes walking in travel targets scenario

The same equation applies for cycling. Speed per hour is the corresponding values for the MET values for walking and cycling from the physical activity compendium (Ainsworth et al., 2011) (Table E).

Table E Active transport speed

|  | **Baseline** | **Sensitivity** |
| --- | --- | --- |
|  | **km/h lower^c^** | **km/h upper** |
| Walk^a^ | 4.48 | 5.12 |
| Bicycle^b^ | 16 | 19.04 |

a. Walking for pleasure in the PA compendium (3.5 MET-minutes). b. Bicycling, to/from work, self-selected pace; bicycling (6.8 MET-minutes). c. Values in source are reported per miles

For additional walking of public transport trips (Table 6 in the main manuscript), the following formula was applied, by age and sex:

$$\boldsymbol{\Delta walking mins. of PT}\boldsymbol{=}\boldsymbol{\Delta PT trips per day*Mean wal}\boldsymbol{king in PT trips*5}$$

Equation C Additional minutes walking for public transport trips in travel targets scenario

### PM_2.5_

With the help of the statistical software Stata (*StataCorp, 2013.Stata Statistical Software: Release 13. College Station, TX: StataCorp LP)*  we derived mean background PM_2.5_ measured in each of the monitoring sites and estimated the arithmetic mean of all sites with more than 75% of observation in a year (Table F) (Queensland Goverment, 2015). Our overall estimated mean of 6.96 (SE 0.02) µg/m^3^ was decreased in the travel targets scenario proportionally to the change in the contribution to ambient PM_2.5_ from passenger cars and buses (Table 8 main manuscript).

Table F Level of data completeness (%) by monitoring site and estimated annual mean for background PM_2.5_ (µg/m^3^)

| **Year** | **Rocklea** | | **South Brisbane** | | **Woolloongabba** | | **Wynnum North** | | **Lutwyche** | |
| --- | --- | --- | --- | --- | --- | --- | --- | --- | --- | --- |
|  | **%** | **Mean** | **%** | **Mean** | **%** | **Mean** | **%** | **Mean** | **%** | **Mean** |
| 2006 | 90 | 6.23 | N/A | N/A | N/A | N/A | N/A | N/A | N/A | N/A |
| 2007 | 93 | 6.77 | N/A | N/A | N/A | N/A | N/A | N/A | N/A | N/A |
| 2008 | 85 | 5.61 | N/A | N/A | 54 | 9.27 | 39 | 4.93 | N/A | N/A |
| 2009 | 90 | 10.72 | 75 | 10.79 | 95 | 8.61 | 94 | 5.66 | N/A | N/A |
| 2010 | 97 | 8.25 | 84 | 6.84 | 95 | 8.30 | 89 | 4.30 | N/A | N/A |
| 2011 | *3* | 6.66 | 99 | 7.04 | 95 | 8.68 | N/A | N/A | 67 | 10.42 |
| 2012 | *56* | 6.93 | 95 | 6.93 | 89 | 7.78 | N/A | N/A | 46 | 5.51 |
| 2013 | 86 | 6.57 | 94 | 7.84 | 93 | 8.03 | 91 | 4.76 | N/A | N/A |

N/A=not available

As described in the main manuscript, we incorporated the health effects of exposure to PM_2.5_ via two mechanisms: the societal and individual effects. For both effects we used the relative risks presented in Table G.

Table G Relative Risks for exposure to PM_2.5_

| **Outcome (Mortality)** | **RR** | **Exposure change** |
| --- | --- | --- |
| Cardiovascular (WHO 2014) ^a^ | 1.10 (1.05, 1.15) | 10 μg/m^3^ |
| Respiratory (WHO 2014)^b^ | 1.10 (0.98, 1.24) | 10 μg/m^3^ |
| Lung cancer (Hamra et al., 2014) | 1.09 (1.04, 1.14) | 10 μg/m^3^ |

a. Used for IHD and ischemic stroke. b. Used for chronic obstructive pulmonary disease

The following formula was used to calculate the relative risks for the travel targets scenario:

$$\mathrm{RR}_{travel targets}=exp \left( \frac{\ln\left( {RR}_{2.5} \right)*Change in exposure}{10} \right)$$

Equation D RR travel targets

Change in exposure for the societal effect was estimated as the difference in the mean PM_2.5_ between baseline and travel targets scenario (Table 8 main manuscript).

A similar formula as with the societal effect was used for the calculation of the relative risk, however, the calculaltion of the change in exposure differs. Following procedures developed in past studies (Rojas-Rueda et al., 2012; Tainio et al., 2016; World Health Organization, 2014), we calculated the change in exposure as follows:

$$\Delta individual exposure= \left( \left( Total dose travel targets/Total dose baseline \right)-1 \right)*background exposure$$

Equation E Change in individual exposure to PM_2.5_

In the MSLT model we multiplied estimated relative risks for the travel targets scenario by incidence of IHD, ischemic stroke, chronic obstructive pulmonary disease and tracheal, bronchus and lung cancers.

Please note that in the original document by the WHO the term used is “equivalent change”. Here we just use ‘change’ as we found this terminology confusing.

To estimate the total dose per week for the baseline (status quo) and travel targets scenario we needed activity-specific information for: hours per week spent, ventilation rates, concentration of PM_2.5_  and total inhaled dose of PM_2.5_ (Table H example for males aged 17 to 49). We assumed that people sleep eight hours per day and spend the rest of the day in activities (other activities) that have a ventilation rate equivalent to resting and are exposed to average background PM_2.5_. The only difference between scenarios is that car occupant time at baseline is replaced by active transport for the travel targets scenario (hrs/wk. in activity in Table H). Using ventilation rates per minute and concentration of PM_2.5_ for each of the activites previously applied in a similar study (Tainio et al., 2016) we calculated the total inhaled dose per week for each activity. Sleep and resting time have the equivalent to the background PM_2.5_ concentration, whereas for the rest of the activities have higher concentration levels.

Table H Parameters and calculations for the change in exposure at the individual level for the travel targets scenario

| **Scenario** | **Activity** | **Hrs/wk. in activity** | **Ventilation rates (m^3^/hr)** | **Concentration PM_2.5_ (µg/m3)^a^** | **Inhaled dose PM_2.5_ (µg/m^3^)^c^** | **Total dose per week** |
| --- | --- | --- | --- | --- | --- | --- |
| **Baseline** | **Sleep** | 56 | 0.27 | 6.96^b^ | 105.20 |  |
|  | ***Car Occupant*** | *0.75* | 0.61 | 8.35 | 3.84 |  |
|  | **Other activities** | 111.25 | 0.61 | 6.96 | 472.14 | 581.18 |
| **Travel targets** | **Sleep** | 56 | 0.27 | 6.96 | 105.20 |  |
|  | **Other activities** | 111.25 | 0.61 | 6.96 | 472.14 |  |
|  | ***Walk*** | *0.22* | 1.37 | 7.65 | 2.36 |  |
|  | ***Cycle*** | *0.27* | 2.55 | 13.92 | 9.47 |  |
|  | ***PT (walking)*** | *0.26* | 1.37 | 7.65 | 2.75 | 591.92 |

a. Car occupant (1.2), walking (1.1) and bicycling (2) (Tainio et al., 2016). b. Note that this includes the reduction of background PM_2.5_ from achieving the travel targets. c. Inhaled dose PM_2.5_=Hrs per week in activity*ventilation rates*Concentration

### Road trauma

A simple distance based model was used to model the effect of road trauma of achieving the travel targets scenario. Firstly, baseline injuries (hospitalisations and medically treated) and fatalities were summarised by combinations of victim and striking mode, using data from the Queensland Road Crash Database maintained by Transport and Main Roads (2014) (Table I and J). The database provides information on the unit counts for modes involved in a crash. However, there is no indication of striking and victim modes involved. We therefore assumed than in a two modes crash, the largest was the striking vehicle. When more than two modes were involved in a crash, the largest was assumed the striking vehicle and the rest victims. Road crashes only involving one mode are presented as “Single mode”. A fatality is a person who dies within 30 days as a result of injuries sustained in a road traffic crash. A hospitalised causality is a person who is transported to hospital as a result of a road traffic crash who does not die from injuries sustained in the crash within 30 days of the crash (TMR 2012). A medically treated casualty is a person requiring medical treatment (but not hospitalisation) as a result of a road traffic crash (TMR 2012).

Table I Baseline road fatalities

|  | **Striking** | | | | | | | **Single vehicle** | **Total** |
| --- | --- | --- | --- | --- | --- | --- | --- | --- | --- |
| **Victim** | **Pedestrian** | **Cyclist** | **Motorcycle** | **Car** | **Bus** | **Truck** | **Other** | **Victim mode** |  |
| **Pedestrian** | 0 | 0 | 0 | 2 | 1 | 0 | 0 | 0 | 3 |
| **Cyclist** | 0 | 0 | 0 | 1 | 0 | 0 | 1 | 1 | 3 |
| **Motorcycle** | 0 | 0 | 0 | 5 | 0 | 2 | 0 | 4 | 11 |
| **Car** | 0 | 0 | 0 | 0 | 0 | 3 | 0 | 8 | 11 |
| **Bus** | 0 | 0 | 0 | 0 | 0 |  | 0 | 0 | 0 |
| **Truck** | 0 | 0 | 0 | 0 | 0 | 1 | 0 | 1 | 2 |
| **Other** | 0 | 0 | 0 | 0 | 0 | 0 | 0 | 0 | 0 |
| **Total** | 0 | 0 | 0 | 8 | 1 | 6 | 1 | 14 | 30 |

Table J Baseline road injuries

|  | **Striking** | | | | | | | **Single vehicle** | **Total** |
| --- | --- | --- | --- | --- | --- | --- | --- | --- | --- |
| **Victim** | **Pedestrian** | **Cyclist** | **Motorcycle** | **Car** | **Bus** | **Truck** | **Other** | **Victim mode** |  |
| **Pedestrian** | 0 | 2 | 17 | 216 | 16 | 12 | 0 | 0 | 263 |
| **Cyclist** | 0 | 2 | 2 | 186 | 4 | 9 | 1 | 17 | 221 |
| **Motorcycle** | 0 | 0 | 3 | 303 | 7 | 13 | 2 | 103 | 431 |
| **Car** | 0 | 0 | 0 | 2105 | 67 | 273 | 28 | 412 | 2885 |
| **Bus** | 0 | 0 | 0 | 0 | 4 | 0 | 0 | 19 | 23 |
| **Truck** | 0 | 0 | 0 | 0 | 0 | 4 | 3 | 23 | 30 |
| **Other** | 0 | 0 | 0 | 0 | 0 | 0 | 0 | 4 | 4 |
| **Total** | 0 | 4 | 22 | 2810 | 98 | 311 | 34 | 578 | **3857** |

We used an application of the Integrated Transport and Health Impact model (ITHIM) for the calculation of baseline injury and fatality rates per pairwise combination of victim and striking mode (Maizlish et al., 2013; Woodcock et al., 2013). Calculating baseline rates per pairwise combination requires data on the number of injuries/fatalities, person-kilometres travelled for the victim mode, and vehicle-kilometres travelled for the striking mode per year (Equation F). Person-kilometres travelled were derived from the South East Queensland Household Travel Survey (TMR 2009) for pedestrians, bicyclists and cars (Table K). For car occupants, motorcycle/moped, bus, trucks and other modes, vehicle- kilometres travelled were those reported in the Survey of Motor Vehicle Use (ABS 2011). For pedestrians and bicyclist person- kilometres travelled are the same as vehicle kilometres travelled. For motorcycle/moped, vehicle-kilometres travelled were the same as person-kilometres travelled. Number of injuries and fatalities for the travel targets scenario were estimated using Equation G.

$$R_{0}=\frac{{Number of injuries}_{Victim}}{{({PKM}_{Victim}*{VKT}_{Striking vehicle})}^{0.5}}$$

Equation F Rate of injuries per pairwise combination of victim and striking modes

$${{Number of injuries}_{{Victim}_{0}}=R}_{0}*{({PKM}_{Victim}*{VKT}_{Striking vehicle})}^{0.5}$$

Equation G Number of injuries per pairwise combination of victim and striking modes

Table K Person and vehicle kilometres travelled per capita for baseline and travel targets scenario

|  | **Baseline** | | **Travel targets scenario** | |
| --- | --- | --- | --- | --- |
| **Mode** | **Person** | **Vehicles** | **Person** | **Vehicle** |
| **Pedestrian** | 108 | 108 | 197 | 197 |
| **Cyclist** | 69 | 69 | 157 | 157 |
| **Car occupant** | 10,050 | 8,200 | 9,555 | 7,795.57 |
| **Motorcycle** | 5,100 | 5,100 | 5,100 | 5,100 |
| **Bus** | 24,000 | 24,000 | 24,000 | 28,038 |
| **Truck** | 28,800 | 28,800 | 28,800 | 28,800 |
| **Others^a^** | 36,400 | 36,400 | 36,400 | 36,400 |

a. Light commercial vehicles

## He**alth care costs and costs in added life years**

In tables L and M we present health care costs and cost in added life years used in the MSLT.

Table L Disease cost per case

| Age and sex | Ischemic heart disease^a^ | Stroke^a^ | Type 2 diabetes^a^ | Breast Cancer^b^ | Colon  cancer^b^ | Lung, tracheal and bronchus cancer^b^ | COPD^a^ | Road traffic injuries^c^ |
| --- | --- | --- | --- | --- | --- | --- | --- | --- |
| **Male** |  |  |  |  |  |  |  |  |
| 24-34 |  |  |  |  |  |  |  | $65,414 |
| 34-44 |  |  |  |  |  |  |  | $22,776 |
| 44-55/>55 | $9,007 | $10,056 | $613 | - | $20,354 | $19,962 | $2,064 | $14,574 |
| 55–64 | $8,309 | $13,459 | $974 | - | $23,030 | $19,682 | $2,064 | $13,769 |
| 65–74 | $7,243 | $18,270 | $1,175 | - | $23,348 | $20,221 | $2,064 | $15,184 |
| 75+ | $8,384 | $24,047 | $1,596 | - | $25,213 | $19,210 | $2,064 | $37,576 |
| **Female** |  |  |  |  |  |  |  |  |
| 24-34 |  |  |  |  |  |  |  | $47,890 |
| 34-44 |  |  |  |  |  |  |  | $18,150 |
| 44-55/>55 | $6,021 | $6,848 | $537 | $16,365 | $21,121 | $23,489 | $2,064 | $13,236 |
| 55–64 | $7,209 | $9,225 | $938 | $15,064 | $20,252 | $21,124 | $2,064 | $12,219 |
| 65–74 | $8,041 | $11,664 | $1,118 | $16,223 | $22,130 | $22,725 | $2,064 | $16,337 |
| 75+ | $8,052 | $33,981 | $1,596 | $17,336 | $22,578 | $21,222 | $2,064 | $30,913 |

a. Cost per prevalent case of disease. b. Cost per incident case of disease. c. Road traffic injuries costs are per prevalent year lived with disability.

N.B. Costs are in Australian dollars, from the Disease Costs and Impact Study 2001 prepared by the Australian Institute of Health and Welfare and indexed to the year 2013 using the health price index (Australian Institute of Health and Welfare, 2001) for all diseases except COPD. COPD costs are estimates from the AIHW for 2008-2009 without disaggregation by age and sex (2016b). Incidence, prevalence and YLDs are for 2000 and 2010 (COPD) from the GBD 2015 study (Institute for Health Metrics and Evaluation (IHME), 2016).

Table M Costs for all other diseases in added life years

| **Age and sex** | **Cost of all other diseases** |
| --- | --- |
| **Male** |  |
| 15–24 | $1,792 |
| 25–34 | $1,750 |
| 35–44 | $1,925 |
| 45–54 | $2,474 |
| 55–64 | $3,867 |
| 65–74 | $6,702 |
| 75-84 | $10,758 |
| 85+ | $17,537 |
| **Female** |  |
| 15–24 | $2,429 |
| 25–34 | $2,984 |
| 35–44 | $2,698 |
| 45–54 | $3,186 |
| 55–64 | $4,365 |
| 65–74 | $6,826 |
| 75-84 | $10,974 |
| 85+ | $20,563 |

N.B. Costs are in Australian dollars, from the Disease Costs and Impact Study 2001 prepared by the Australian Institute of Health and Welfare and indexed to the year 2013 using the health price index (Australian Institute of Health and Welfare, 2001). Includes overall health care costs per person minus costs of diseases and injuries included in the model (Table S12).

# Sensitivity analysis

Table N is a summary of sensitivity analyses described in the main manuscript.

Table N Univariate sensitivity analysis parameters

| **Parameter** | | **Base case** | | **Sensitivity** |
| --- | --- | --- | --- | --- |
| **Baseline and travel targets scenario** | | | | |
| Discount rate health outcomes and health care costs | 0% health and 3% health care costs per annum/3% health and 3% health care costs per annum | | 3% health and 5% health care costs per annum (Gold, 1996; Tan-Torres Edejer, 2003) | |
| **Travel targets scenario** | | | | |
| Time taken per km walked and cycled^a^ | Walking 4.48 km/h, cycling 16 km/h | | Walking 5.12km/h, cycling 19.04km/h (upper limits from PA compendium (Ainsworth et al., 2011) | |
| PM_2.5_ source apportionment | 18% | | 7% and 30% (Friend et al., 2011) | |
| Passenger vehicles and buses contribution to traffic related PM_2.5_ | 28% and 10% | | 65% passenger vehicles | |
| Bicycling exposure to PM_2.5_ | 2 relative to ambient PM_2.5_ | | 1.1 relative to ambient PM_2.5_ (assumption, same as walking) | |
| Road trauma-km travelled | Non-linear | | Linear | |

a. Distances per km walked and cycled are based on the PA compendium’s lower value for the corresponding MET-rates. MET are the energy expenditure of an activity compared to resting.

# Additional results to the main manuscript

In Table O we present results for the sensitivity scenarios discussed in the main manuscript.

Table O Health care costs and health outcomes for sensitivity scenarios (95% uncertainty interval)

|  | **Health adjusted life years (thousand)** | **Life years (thousand)** | **Health care costs total (millions)^a^** | **Other health care costs in added Lys total (millions)** |
| --- | --- | --- | --- | --- |
| 1. Walking speed 5.12km/h and cycling 19.4km/h**^a^** | 30.3  (17.7 to 44.2) | 26.3  (11.6 to 41.8) | -$294  (-$442 to -$159) | $122  ($43 to $204) |
| 2. PM_2.5_ source apportionment high | 32.8  (19.8 to 47.0) | 28.3  (13.3 to 44.3) | -$313  (-$464 to -$174) | $130  ($49 to $214) |
| 3. PM2.5 source apportionment low | 32.5  (19.5 to 46.7) | 28.1  (13.1 to 43.9) | -$312  (-$463 to -$173) | $129  ($48 to $213) |
| 4. Passenger vehicles PM_2.5_ attribution high^c^ | 33.0  (20.1 to 47.1) | 28.5  (13.5 to 44.5) | -$315  (-$465 to -$176) | $131  ($50 to $215) |
| 5. Cycling exposure to PM^2.5^ similar to walking | 32.7  (19.8 to 46.9) | 28.3  (13.3 to 44.2) | -$313  (-$463 to -$174) | $129  ($49 to $213) |
| 6. Linear association road trauma | 21.6  (8.6 to 35.8) | 19.0  (4.1 to 34.9) | -$264  (-$415 to -$125) | $093  ($12 to $177) |
| 7. Discount health 3% | 10.0  (6.0 to 14.3) | 7.8  (3.6 to 12.3) | -$312  (-$463 to -$173) | $43  ($14 to $74) |
| 8. Discount health care costs 5% | 32.6  (19.6 to 46.8) | 28.1  (13.1 to 44.0) | -$175  (-$257 to -$099) | $61  ($21 to $103) |
| 9. Discount health 3% and health care costs 3% | 10.0  (6.0 to 14.3) | 7.8  (3.6 to 12.3) | -$312  (-$463 to -$173) | $43  ($14 to $74) |

a. Negative values are savings. b. ***Base case:*** Walking speed of 4.48 km/h and cycling speed of 16 km/h. c. Base case 28% and sensitivity scenario 65%.

Intermediate outputs used to modify incidence of physical activity and PM_2.5_ related diseases are presented in tables P and Q.

Table P Population impact fraction for PA-related diseases for travel targets scenario compared to baseline by age and sex used to modify incidence rates in MSLT^a^

| **Age and sex** | **IHD** | **Ischemic stroke** | **Type 2 diabetes** | **Colon**  **cancer** | **Breast cancer** |
| --- | --- | --- | --- | --- | --- |
| 20-25, male | 0.04 | 0.05 | 0.04 | 0.07 |  |
| 25-29, male | 0.05 | 0.06 | 0.05 | 0.08 |  |
| 30-34, male | 0.05 | 0.07 | 0.05 | 0.08 |  |
| 35-39, male | 0.06 | 0.07 | 0.05 | 0.09 |  |
| 40-44, male | 0.06 | 0.07 | 0.05 | 0.09 |  |
| 45-49, male | 0.06 | 0.08 | 0.06 | 0.10 |  |
| 50-54, male | 0.05 | 0.07 | 0.04 | 0.08 |  |
| 55-59, male | 0.05 | 0.07 | 0.04 | 0.09 |  |
| 60-64, male | 0.04 | 0.06 | 0.04 | 0.08 |  |
| 65-69, male | 0.05 | 0.07 | 0.04 | 0.09 |  |
| 70-74, male | 0.04 | 0.07 | 0.04 | 0.08 |  |
| 75-79, male | 0.02 | 0.05 | 0.02 | 0.06 |  |
| 80-84, male | 0.02 | 0.04 | 0.02 | 0.05 |  |
| 85-89, male | 0.02 | 0.05 | 0.02 | 0.05 |  |
| 90-94, male | 0.02 | 0.05 | 0.02 | 0.05 |  |
| 95-100, male | 0.02 | 0.05 | 0.02 | 0.05 |  |
| 20-25, female | 0.04 | 0.04 | 0.03 | 0.05 | 0.03 |
| 25-29, female | 0.04 | 0.05 | 0.04 | 0.06 | 0.03 |
| 30-34, female | 0.04 | 0.05 | 0.04 | 0.07 | 0.03 |
| 35-39, female | 0.04 | 0.06 | 0.04 | 0.07 | 0.03 |
| 40-44, female | 0.05 | 0.07 | 0.05 | 0.08 | 0.03 |
| 45-49, female | 0.05 | 0.07 | 0.05 | 0.09 | 0.04 |
| 50-54, female | 0.04 | 0.06 | 0.03 | 0.07 | 0.03 |
| 55-59, female | 0.03 | 0.05 | 0.03 | 0.07 | 0.03 |
| 60-64, female | 0.04 | 0.06 | 0.03 | 0.08 | 0.03 |
| 65-69, female | 0.03 | 0.05 | 0.03 | 0.07 | 0.02 |
| 70-74, female | 0.03 | 0.06 | 0.03 | 0.07 | 0.03 |
| 75-79, female | 0.03 | 0.06 | 0.03 | 0.08 | 0.03 |
| 80-84, female | 0.02 | 0.05 | 0.02 | 0.06 | 0.02 |
| 85-89, female | 0.03 | 0.06 | 0.03 | 0.07 | 0.03 |
| 90-94, female | 0.03 | 0.06 | 0.03 | 0.07 | 0.03 |
| 95-100, female | 0.03 | 0.06 | 0.03 | 0.07 | 0.03 |

a. Only for baseline scenario. Incidence was modified by 1-PIF.

Table Q Relative risks for PM_2.5_-related diseases for travel targets scenario compared to baseline by age and sex used to modify incidence rates in MSLT

| **Age and sex** | **COPD soc. effect^a^** | **TBL cancers^b^ soc. effect.** | **IHD soc. effect** | **Ischemic stroke soc. effect** | **COPD ind. Effect^c^** | **TBL cancers ind. effect.** | **IHD ind. effect** | **Ischemic stroke ind. effect** |
| --- | --- | --- | --- | --- | --- | --- | --- | --- |
| 20-25, male | 0.9999 | 0.9999 | 0.9999 | 0.9999 | 1.0012 | 1.0011 | 1.0012 | 1.0012 |
| 25-29, male | 0.9999 | 0.9999 | 0.9999 | 0.9999 | 1.0012 | 1.0011 | 1.0012 | 1.0012 |
| 30-34, male | 0.9999 | 0.9999 | 0.9999 | 0.9999 | 1.0012 | 1.0011 | 1.0012 | 1.0012 |
| 35-39, male | 0.9999 | 0.9999 | 0.9999 | 0.9999 | 1.0012 | 1.0011 | 1.0012 | 1.0012 |
| 40-44, male | 0.9999 | 0.9999 | 0.9999 | 0.9999 | 1.0012 | 1.0011 | 1.0012 | 1.0012 |
| 45-49, male | 0.9999 | 0.9999 | 0.9999 | 0.9999 | 1.0012 | 1.0011 | 1.0012 | 1.0012 |
| 50-54, male | 0.9999 | 0.9999 | 0.9999 | 0.9999 | 1.0008 | 1.0007 | 1.0008 | 1.0008 |
| 55-59, male | 0.9999 | 0.9999 | 0.9999 | 0.9999 | 1.0008 | 1.0007 | 1.0008 | 1.0008 |
| 60-64, male | 0.9999 | 0.9999 | 0.9999 | 0.9999 | 1.0008 | 1.0007 | 1.0008 | 1.0008 |
| 65-69, male | 0.9999 | 0.9999 | 0.9999 | 0.9999 | 1.0008 | 1.0007 | 1.0008 | 1.0008 |
| 70-74, male | 0.9999 | 0.9999 | 0.9999 | 0.9999 | 1.0008 | 1.0007 | 1.0008 | 1.0008 |
| 75-79, male | 0.9999 | 0.9999 | 0.9999 | 0.9999 | 1.0001 | 1.0001 | 1.0001 | 1.0001 |
| 80-84, male | 0.9999 | 0.9999 | 0.9999 | 0.9999 | 1.0001 | 1.0001 | 1.0001 | 1.0001 |
| 85-89, male | 0.9999 | 0.9999 | 0.9999 | 0.9999 | 1.0001 | 1.0001 | 1.0001 | 1.0001 |
| 90-94, male | 0.9999 | 0.9999 | 0.9999 | 0.9999 | 1.0001 | 1.0001 | 1.0001 | 1.0001 |
| 95-100, male | 0.9999 | 0.9999 | 0.9999 | 0.9999 | 1.0001 | 1.0001 | 1.0001 | 1.0001 |
| 20-25, female | 0.9999 | 0.9999 | 0.9999 | 0.9999 | 1.0007 | 1.0006 | 1.0007 | 1.0007 |
| 25-29, female | 0.9999 | 0.9999 | 0.9999 | 0.9999 | 1.0007 | 1.0006 | 1.0007 | 1.0007 |
| 30-34, female | 0.9999 | 0.9999 | 0.9999 | 0.9999 | 1.0007 | 1.0006 | 1.0007 | 1.0007 |
| 35-39, female | 0.9999 | 0.9999 | 0.9999 | 0.9999 | 1.0007 | 1.0006 | 1.0007 | 1.0007 |
| 40-44, female | 0.9999 | 0.9999 | 0.9999 | 0.9999 | 1.0007 | 1.0006 | 1.0007 | 1.0007 |
| 45-49, female | 0.9999 | 0.9999 | 0.9999 | 0.9999 | 1.0007 | 1.0006 | 1.0007 | 1.0007 |
| 50-54, female | 0.9999 | 0.9999 | 0.9999 | 0.9999 | 1.0003 | 1.0003 | 1.0003 | 1.0003 |
| 55-59, female | 0.9999 | 0.9999 | 0.9999 | 0.9999 | 1.0003 | 1.0003 | 1.0003 | 1.0003 |
| 60-64, female | 0.9999 | 0.9999 | 0.9999 | 0.9999 | 1.0003 | 1.0003 | 1.0003 | 1.0003 |
| 65-69, female | 0.9999 | 0.9999 | 0.9999 | 0.9999 | 1.0003 | 1.0003 | 1.0003 | 1.0003 |
| 70-74, female | 0.9999 | 0.9999 | 0.9999 | 0.9999 | 1.0003 | 1.0003 | 1.0003 | 1.0003 |
| 75-79, female | 0.9999 | 0.9999 | 0.9999 | 0.9999 | 1.0002 | 1.0003 | 1.0002 | 1.0003 |
| 80-84, female | 0.9999 | 0.9999 | 0.9999 | 0.9999 | 1.0002 | 1.0003 | 1.0002 | 1.0003 |
| 85-89, female | 0.9999 | 0.9999 | 0.9999 | 0.9999 | 1.0002 | 1.0003 | 1.0002 | 1.0003 |
| 90-94, female | 0.9999 | 0.9999 | 0.9999 | 0.9999 | 1.0002 | 1.0003 | 1.0002 | 1.0003 |
| 95-100, female | 0.9999 | 0.9999 | 0.9999 | 0.9999 | 1.0002 | 1.0003 | 1.0002 | 1.0003 |

a. Societal effect. b. Tracheal, bronchus and lung cancer cancers TBL. **c**. Individual effect.

# Validation tests

We conducted a number of tests to check for any inconsistencies in the MSLT (Table R). Excluding all risk factors results in zero changes. Applying an extreme discount rate of 20% to health and health care costs significantly reduces the outcomes.

Table R Model tests^a^

|  | **HALYs** | **LYs** | **Savings in health care costs** | **Health care costs of added LYs** |
| --- | --- | --- | --- | --- |
| Complete model | 33,455 | 28,723 | -$ 319,094,510 | $ 131,530,927 |
| Exclude all risk factors | - | - | - | - |
| Discount health care costs at 20% | 36,725 | 32,447 | -$ 11,387,186 | $ 1,898,921 |
| Discount health at 20% | 166 | 105 | -$ 319,094,510 | $ 1,237,623 |

a. Results from deterministic analysis.

**References**

Ainsworth, et al. (2011). 2011 Compendium of Physical Activities: a second update of codes and MET values. *Med Sci Sports Exerc, 43*(8), 1575-1581. doi:10.1249/MSS.0b013e31821ece12

Asia Pacific Cohort Studies Collaboration. (2003). The Effects of Diabetes on the Risks of Major Cardiovascular Diseases and Death in the Asia-Pacific Region. *Diabetes Care, 26*(2), 360-366. doi:10.2337/diacare.26.2.360

Australian Bureau of Statistics. (2011). Survey of Motor Vehicle Use, Australia, 12 months ended 31 October 2010. Canberra.

Australian Bureau of Statistics. (2015). Australian Health Survey: Physical Activity, 2011-12. Retrieved from <http://www.abs.gov.au/ausstats/abs@.nsf/Lookup/D4495467B7F7EB01CA257BAC0015F593?opendocument>

Australian Bureau of Statistics. (2016a). *Deaths, Australia, 2014*. Retrieved from: <http://www.abs.gov.au/ausstats/abs@.nsf/mf/3302.0>

Australian Bureau of Statistics. (2016b). *Estimated Resident Population By Single Year Of Age, Australia*. Table 59. Retrieved from: <http://www.abs.gov.au/AUSSTATS/abs@.nsf/DetailsPage/3101.0Dec%202015?OpenDocument>

Australian Institute of Health and Welfare. (2001). *Disease costs and impact study data*. Retrieved from

Australian Institute of Health and Welfare. (2016a). Australian Cancer Incidence and Mortality (ACIM) books. Canberra.

Australian Institute of Health and Welfare. (2016b). How much is spent on COPD? Retrieved from <http://www.aihw.gov.au/copd/expenditure/>

Barendregt. (2012). EpiGear International. Retrieved from <http://www.epigear.com/index_files/prevent.html>

Barendregt, et al. (2003). A generic model for the assessment of disease epidemiology: the computational basis of DisMod II. *Population Health Metrics, 1*(1), 4-4. doi:10.1186/1478-7954-1-4

Barendregt, & Veerman. (2010). Categorical versus continuous risk factors and the calculation of potential impact fractions. *Journal of Epidemiology and Community Health, 64*(3), 209-212. doi:10.1136/jech.2009.090274

Cobiac, et al. (2009). Cost-effectiveness of interventions to promote physical activity: a modelling study. *Plos Medicine, 6*(7), e1000110-e1000110. doi:10.1371/journal.pmed.1000110

Danaei, et al. (2009). The preventable causes of death in the United States: comparative risk assessment of dietary, lifestyle, and metabolic risk factors. *PLoS Medicine, 6*(4), e1000058. doi:10.1371/journal.pmed.1000058

Department of Transport and Main Roads (TMR). (2009). *2009 South East Queensland Household Travel Survey*. Retrieved from: <https://data.qld.gov.au/dataset/2009-south-east-queensland-household-travel-survey>

Department of Transport and Main Roads (TMR). (2012). *Fatal Road Traffic Crashes in Queensland 2011*. Retrieved from

Department of Transport and Main Roads (TMR). (2014). Crash data from Queensland roads. Retrieved from <https://data.qld.gov.au/dataset/crash-data-from-queensland-roads>

Friend, et al. (2011). Source apportionment of PM at two receptor sites in Brisbane, Australia. *Environmental Chemistry, 8*(6), 569-580. doi:10.1071/EN11056

GBD 2013 Risk Factors Collaborators. (2015). Global, regional, and national comparative risk assessment of 79 behavioural, environmental and occupational, and metabolic risks or clusters of risks in 188 countries, 1990-2013: A systematic analysis for the Global Burden of Disease Study 2013. *The Lancet, 386*(10010), 2287-2323. doi:10.1016/S0140-6736(15)00128-2

Global Burden of Diseases Study 2013 Collaborators. (2015). Global, regional, and national incidence, prevalence, and years lived with disability for 301 acute and chronic diseases and injuries in 188 countries, 1990–2013: a systematic analysis for the Global Burden of Disease Study 2013. *The Lancet, 386*(9995), 743-800. doi:10.1016/S0140-6736(15)60692-4

Gold. (1996). *Cost-effectiveness in health and medicine*. New York: Oxford University Press.

Hamra, et al. (2014). Outdoor Particulate Matter Exposure and Lung Cancer: A Systematic Review and Meta-Analysis *Environ. Health Perspect.* (Vol. 122, pp. 906-911).

Institute for Health Metrics and Evaluation (IHME). (2015a). GBD Compare.

Institute for Health Metrics and Evaluation (IHME). (2015b). Global Burden of Disease (GBD). Retrieved from <http://www.healthdata.org/gbd>

Institute for Health Metrics and Evaluation (IHME). (2016). Global Burden of Disease Study 2015 (GBD 2015) Results. Retrieved from <http://ghdx.healthdata.org/gbd-results-tool>

Maizlish, et al. (2013). Health Cobenefits and Transportation-Related Reductions in Greenhouse Gas Emissions in the San Francisco Bay Area. *American Journal of Public Health, 103*(4), 703-709. doi:10.2105/AJPH.2012.300939

Queensland Goverment. (2015). *Air Quality Monitoring*. Retrieved from: <https://data.qld.gov.au/dataset?q=air+quality>

Rojas-Rueda, et al. (2012). Replacing car trips by increasing bike and public transport in the greater Barcelona metropolitan area: A health impact assessment study. *Environment International, 49*, 100-109. doi:<http://dx.doi.org/10.1016/j.envint.2012.08.009>

Tainio, et al. (2016). Can air pollution negate the health benefits of cycling and walking? *Preventive medicine, 87*, 233-236. doi:<http://dx.doi.org/10.1016/j.ypmed.2016.02.002>

Tan-Torres Edejer. (2003). *Making choices in health: WHO guide to cost-effectiveness analysis*. Geneva: World Health Organization.

Woodcock, et al. (2013). Health impact modelling of active travel visions for England and Wales using an Integrated Transport and Health Impact Modelling Tool (ITHIM). *PLoS One, 8*(1), e51462. doi:10.1371/journal.pone.0051462

World Health Organization. (2014). *WHO Expert Meeting: Methods and tools for assessing the health risks of air pollution at local, national and international level*. Retrieved from Copenhagen: <http://www.euro.who.int/__data/assets/pdf_file/0010/263629/WHO-Expert-Meeting-Methods-and-tools-for-assessing-the-health-risks-of-air-pollution-at-local,-national-and-international-level.pdf>
